# Comparative metabolomics revealing *Staphylococcus aureus* metabolic response to different antibiotics

**DOI:** 10.1111/1751-7915.12839

**Published:** 2017-08-16

**Authors:** Katie Schelli, Fanyi Zhong, Jiangjiang Zhu

**Affiliations:** ^1^ Department of Chemistry and Biochemistry Miami University 651 E High St. Oxford OH 45056 USA

## Abstract

It is known that changes in bacterial metabolism can contribute to the modulation of bacterial susceptibility to antibiotics. Understanding how bacterial metabolism is impacted by antibiotics may improve our understanding of the antibiotic mechanism of actions from a metabolic perspective. Here, we utilized a mass spectrometry‐based targeted metabolic profiling technique to characterize the metabolome of a pair of isogenic methicillin‐susceptible and resistant *Staphylococcus aureus* (MSSA and MRSA) strains RN450 and 450M treated with the sublethal dose of three antibiotics from different classes (β‐lactams, aminoglycosides and quinolones). These treatments induced a set of metabolic alterations after 6 h of co‐incubation with antibiotics. Similar and divergent metabolic perturbations were observed from different antibiotics to the tested strains. Different metabolic response from MSSA and MRSA to the same antibiotics was also detected in the study and indicated the potentially different stress response mechanism in MSSA and MRSA metabolism. This work has shown that a complex set of metabolic changes can be induced by a variety of antibiotics, and the comparative metabolomics strategy can provide a good understanding of this process from a metabolic perspective.

## Introduction

Antibiotic resistance is a worldwide healthcare problem (Yim *et al*., 2006; Kohanski *et al*., 2010). Taking *Staphylococcus aureus* (*S. aureus*) for example, the Centers for Disease Control and Prevention (CDC) listed methicillin‐resistant *S. aureus* (MRSA) as one of the twelve serious threat‐level pathogens, which killed over 11 000 people in the US in the year 2011 (CDC, 2013). During the past decades, numerous efforts have been made to understand antibiotic resistance mechanisms so that specific treatment strategies can be developed (Sommer and Dantas, 2011; Munck, 2014; Belenky *et al*., 2015). While previous efforts have been made to understand genetic and transcriptional regulatory networks for the antibiotic mechanism of action to *S. aureus*, the post‐treatment metabolic perturbation can also play a major role in understanding the effectiveness of antibiotic killing and inhibition functions. Changes of carbon flux at the cellular level, for example, have been demonstrated to impact antibiotic susceptibility in multiple bacterial species (Brynildsen *et al*., 2013). Additionally, disruption in energy metabolism such as the tricarboxylic acid (TCA) cycle has been reported to increase antibiotic sensitivity both *in vitro* and *in vivo* (Peng *et al*., 2015). Furthermore, metabolic perturbations have been hypothesized to provide a protective state in bacteria by reducing the level of cytotoxic metabolic by‐products, inhibiting antibiotic uptake and/or slowing down cellular growth (Dwyer *et al*., 2014). Therefore, characterizing metabolic changes induced by antibiotics, and understanding how these alterations influence bacterial survivability could be essential steps in addressing the overwhelming problem of antibiotic resistance.

Recent technology developments in system biology, including the progress in multiple ‘omics’ approaches, such as detection and identification of a variety of proteins (using proteomics), and tens of hundreds of metabolites (using metabolomics; Shulaev and Oliver, 2006; Adamski, 2012; Otto *et al*., 2014; Gu *et al*., 2016) vastly enhanced our capability of solving problems from previously poorly researched fields. Here, we propose to use a mass spectrometry (MS)‐based targeted metabolomics approach, for the sensitive, specific and simultaneous detection of more than two hundred metabolites within a single experiment. All detected metabolites have confident identification and therefore are immediately ready for biological interpretation. This MS‐based metabolomics approach has been applied to several studies and has demonstrated strong capability for detecting subtle differences in metabolites from different biological systems (Zhu *et al*., 2014, 2015; Gu *et al*., 2015; Deng *et al*., 2016; Schelli *et al*., 2016; Xu *et al*., 2017; Zhong *et al*., 2017). In this study, we utilized this targeted metabolic profiling platform to broadly and semi‐quantitatively detect massive changes to bacterial metabolism following sublethal dose treatment from three different bactericidal antibiotics: ampicillin, kanamycin and norfloxacin, which were selected from three different antibiotic classes (β‐lactams, aminoglycosides and quinolones). One pair of isogenic *S. aureus* strains was studied to represent the potential difference in methicillin‐susceptible *S. aureus* (MSSA) and MRSA. We found that unique metabolic profiles changes, indicated by alterations of many metabolites and perturbations of important metabolic pathways, were clearly observed from ampicillin‐treated *S. aureus* experiments. Meanwhile, we found that the antibiotic‐induced metabolic alterations were different in MSSA and MRSA for kanamycin and norfloxacin, while the metabolic profile from kanamycin‐treated group showing a clear difference from the untreated group in MRSA experiments, but only displayed a subtle difference in comparison with the untreated group in MSSA. On the other hand, metabolic profile from the norfloxacin‐treated group partially overlapped with the untreated group in MRSA but clearly separated from untreated group in MSSA. Interestingly, we also noticed that the metabolic profiles are naturally different between MSSA and MRSA strains we tested, and after adding a sublethal dose of antibiotics from all three classes we tested, the difference between MSSA and MRSA was dramatically amplified. Taken together, our findings suggested that bactericidal antibiotics can induce a complex set of metabolic changes in *S. aureus* in an antibiotic susceptibility‐dependent manner. Further studies of the different metabolic response from MSSA and MRSA may advance our understanding of the antibiotic mechanism of actions and assist our future decision on the therapeutic method development.

## Results

This study utilized our recently developed targeted metabolic profiling platform, in combination with chemometrics techniques to detect the massive metabolic dysregulation after 6 h' co‐incubation of *S. aureus* to antibiotics. Two isogenic *S. aureus* strains, MSSA RN450 and MRSA 450M, were treated with the sublethal dose of three antibiotics ampicillin, kanamycin and norfloxacin from different classes (β‐lactams, aminoglycosides and quinolones), and the post‐treatment samples were analysed by MS‐based targeted metabolic profiling. Minimum inhibitory concentration (MIC) assays for each antibiotic were determined, and then half MIC concentrations of antibiotic were used for this study to minimize cell death and lysis but at the same time to impact the normal growth of cells. Based on the MIC assays, 0.25 mg l^−1^ ampicillin and kanamycin and 1 mg l^−1^ norfloxacin were used for RN450 strain experiments; for 450M strains, 0.5 mg l^−1^ ampicillin and kanamycin and 2 mg l^−1^ norfloxacin were used in all experiments. The untreated groups were co‐incubated with the same volume of sterilized water and used as control samples.

The targeted metabolic profiling method has been extensively validated by our previous studies (Buas *et al*., 2015, 2017; Zhu *et al*., 2015; Deng *et al*., 2016; Schelli *et al*., 2016; Xu *et al*., 2017) and can screen for 221 metabolites simultaneously with high reproducibility and high throughput. After careful data examination, the average coefficient of variation of 156 detected metabolites (detected in at least 80% of the samples) from quality control samples was at 8.9%, indicating robust analytical performance.

**Figure 1:**
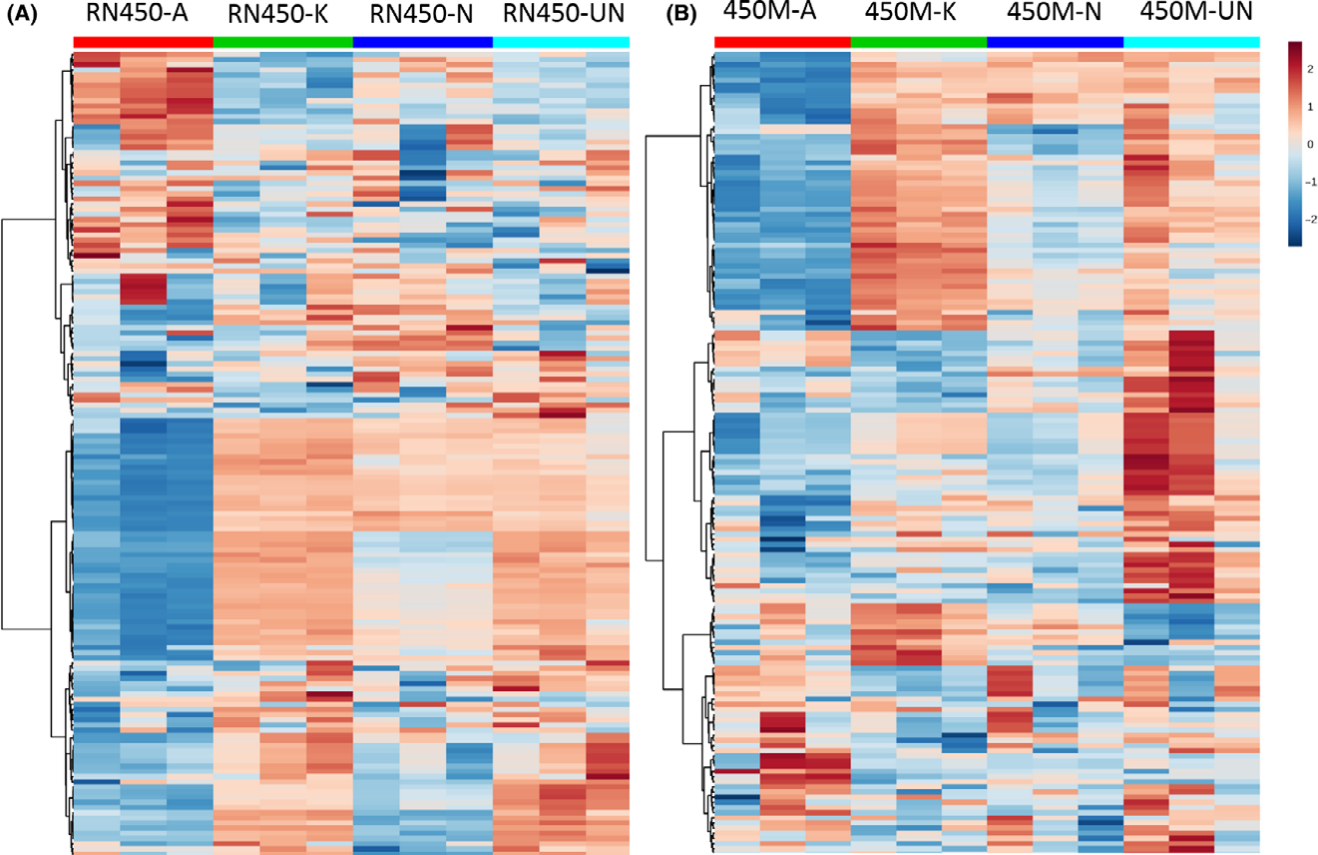
Heatmap presentation of *S. aureus* metabolic changes induced by sublethal dose of antibiotics. A. *S. aureus* RN450 (MSSA) strain dosed with sub‐MIC of ampicillin (RN450‐A), kanamycin (RN450‐K) and norfloxacin (RN450‐N) in comparison with untreated control group (RN450‐UN). B. *S. aureus* 450M (MRSA) strain dosed with sub‐MIC of ampicillin (450M‐A), kanamycin (450M‐K) and norfloxacin (450M‐N) in comparison with untreated control group (450M ‐UN). Each row represents one detected metabolite in the study. Colour difference demonstrated different relative concentration of metabolites across the different experiment groups. Three biological samples from each group were analysed.

We profiled the *S. aureus* metabolome to explore global metabolic alterations induced by ampicillin (A), kanamycin (K) and norfloxacin (N), as well as the untreated (UN) bacterial culture and a pair of heatmaps representing the difference between detected metabolites from three treatments and untreated control groups can be seen in Fig. 1. From the two heatmaps, we can observe that in the RN450 comparison, the RN450‐K group displayed a minor difference in metabolic profiles compared with the untreated group, the subtle difference can also be observed between RN450‐N and RN450‐UN. However, significant changes over a range of metabolites can be seen between metabolic profiles from RN450‐A to RN450‐UN groups. In the case of 450M treatment comparisons, a dramatic decrease in a cluster of metabolites can be observed between the 450M‐A and 450M‐UN, while both 450M‐K and 450M‐N only displayed medium‐to‐minor difference.

**Figure 2:**
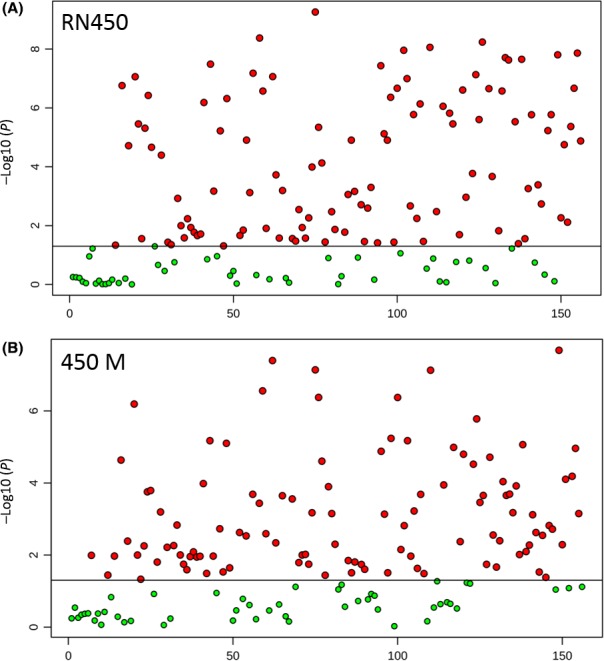
Analysis of variance (ANOVA) plot showing significantly (*P* < 0.05) detected metabolites in comparison with the three antibiotic treatment groups and the untreated control. A. metabolites with significant *P*‐value in RN450 strain experiments. B. metabolites with significant *P*‐value in 450M strain experiments. Each red dot represents one metabolite with *P *<* *0.05 while the green dot represents metabolite without statistical significance. The detailed metabolite identities can be seen in Tables S1 and S2. Three biological samples from each group were analysed.

**Figure 3:**
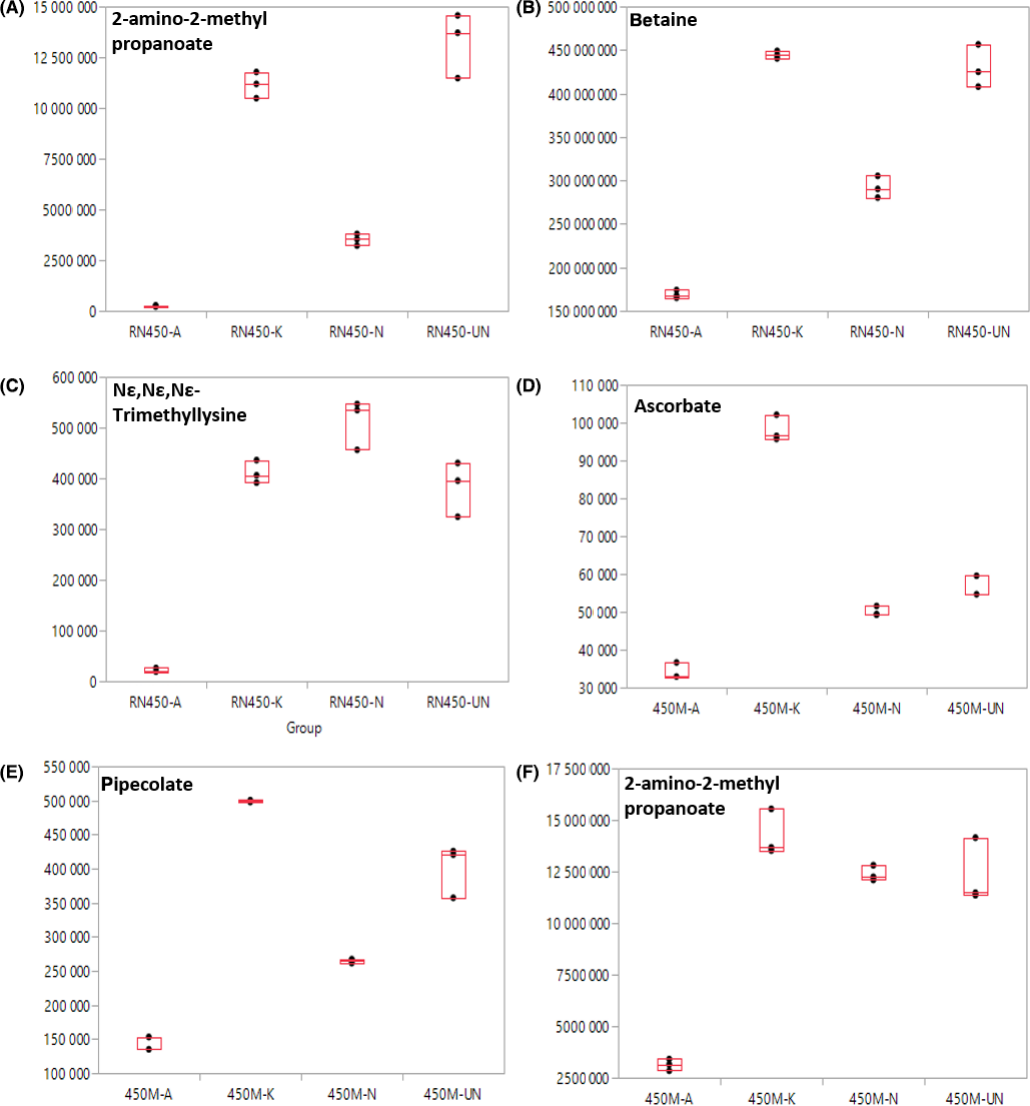
Box plots of representative individual metabolites that have significant *P*‐value by ANOVA when comparing different antibiotic treatment from two *S. aureus* strains. Top three metabolites with significant *P*‐value from RN450 strain (A–C) and 450M strain (D–F) were shown. Three biological samples from each group were analysed.

To further evaluate the metabolic profile differences from different antibiotic treatments, both univariate and multivariate statistical analysis were conducted. Analysis of variance (ANOVA) revealed that 109 metabolites were detected with significance (*P *<* *0.05) from RN450 treatment experiments, and 107 metabolites from 450M treatment experiments were detected with significance based on the ANOVA (Fig. 2). Detailed identities and *P*‐value of metabolites can be seen from supporting information (Tables S1 and S2). Representative metabolites from both ANOVA are plotted in Fig. 3. The three metabolites from Fig. 3A to C, 2‐amino‐2‐methyl propanoate, betaine and Nε, Nε, Nε‐Trimethyllysine were the top three metabolites (smallest *P*‐value) from the RN450 experiments (Table S1) and the three metabolites from Fig. 3D to F, ascorbate, pipecolate and 2‐amino‐2‐methyl propanoate were the top three metabolites from the 450M experiments (Table S2). Interestingly, all six metabolites in both RN450 and 450M experiments indicated that ampicillin‐treated groups produced the lowest level of metabolites in comparison with other three groups, and the difference from ampicillin to untreated group is more significant than the other two antibiotics groups.

**Figure 4:**
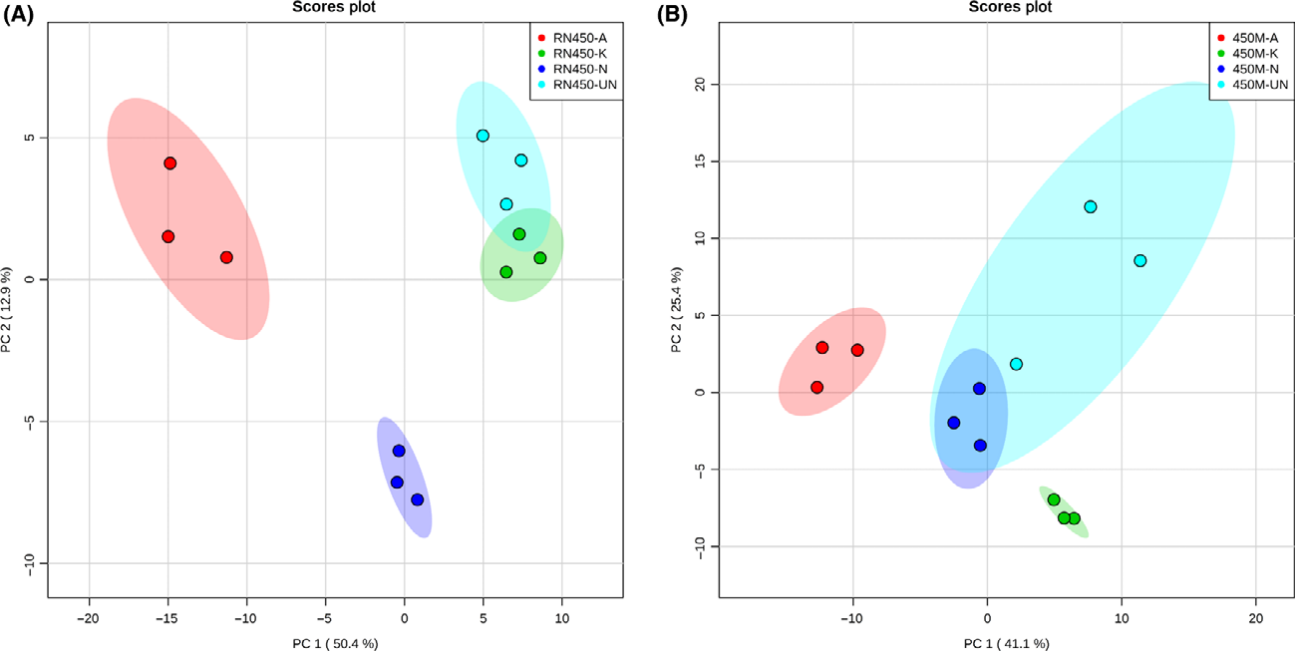
Principal component analysis (PCA) score plot showing distinctive metabolic profiles between *S. aureus* under different antibiotic treatments. A. *S. aureus* RN450 strain dosed with sub‐MIC of ampicillin (RN450‐A), kanamycin (RN450‐K) and norfloxacin (RN450‐N) in comparison with untreated control group (RN450‐UN). B. *S. aureus* 450M strain dosed with sub‐MIC of ampicillin (450M‐A), kanamycin (450M‐K) and norfloxacin (450M‐N) in comparison with untreated control group (450M‐UN). Three biological samples from each group were analysed. The shaded area indicated the 95% confidence region. The corresponding loading plots for each figure are shown in Fig. S1.

**Figure 5:**
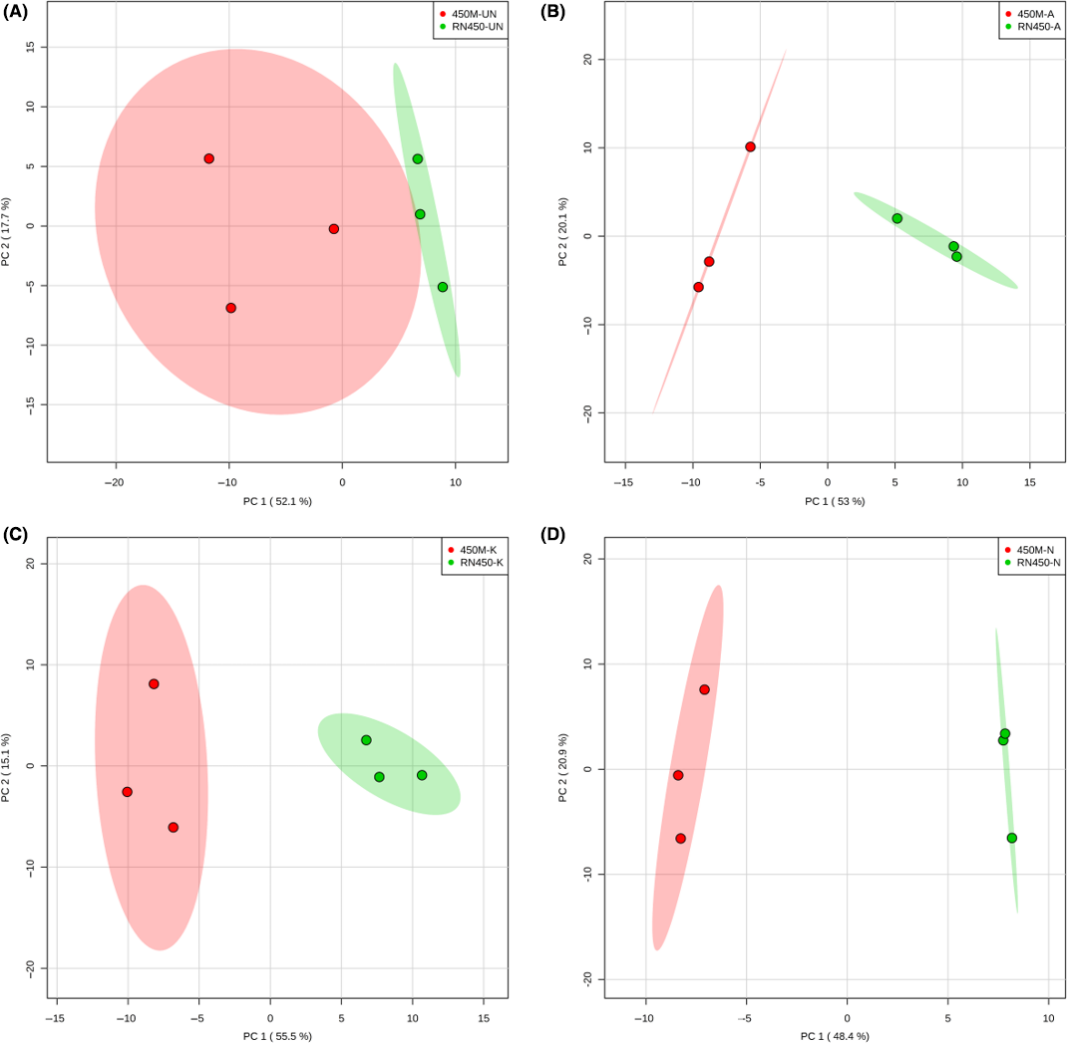
Principal component analysis (PCA) score plot comparing metabolic profiles between methicillin‐susceptible and resistant *S. aureus* (MSSA RN450 and MRSA 450M) from untreated control groups and groups treated with different antibiotics. (A) Untreated controls; (B) ampicillin‐treated groups; (C) kanamycin‐treated groups; (D) norfloxacin‐treated groups. Three biological samples from each group were analysed. The shaded area indicated the 95% confidence region. The corresponding loading plots for each figure are shown in Fig. S2.

Principal component analysis (PCA) was then performed and comparisons were made among the metabolic profiles of three antibiotic‐treated groups and to the control group in each strain (Fig. 4). As can be seen in Fig. 4, on the left panel the ampicillin group is significantly separated from the untreated group as well as the other two antibiotic groups during the RN450 experiments, while the untreated group displayed some level of overlap with kanamycin treatment group due to slightly overlapped metabolic profiles. On the right panel, again the ampicillin‐treated group displayed clear separation away from untreated and the other two antibiotic treatment groups in 450M experiments, while the untreated group largely overlapped with norfloxacin‐treated group. Furthermore, we conducted the pairwise comparison between MSSA and MRSA strains from untreated groups, ampicillin, kanamycin and norfloxacin‐treated groups. As demonstrated in Fig. 5 PCA score plots, while the MSSA and MRSA strains already displayed differentiable metabolic profiles without any treatment, adding a sublethal dose of antibiotics to the bacteria culture induced a dramatic shift of their metabolic profiles, which led to an even greater degree of separation in all three classes of antibiotic tested. These findings again clearly indicated the metabolic alteration difference in antibiotic‐induced stress response from MSSA strain in comparison with MRSA strain.

**Figure 6:**
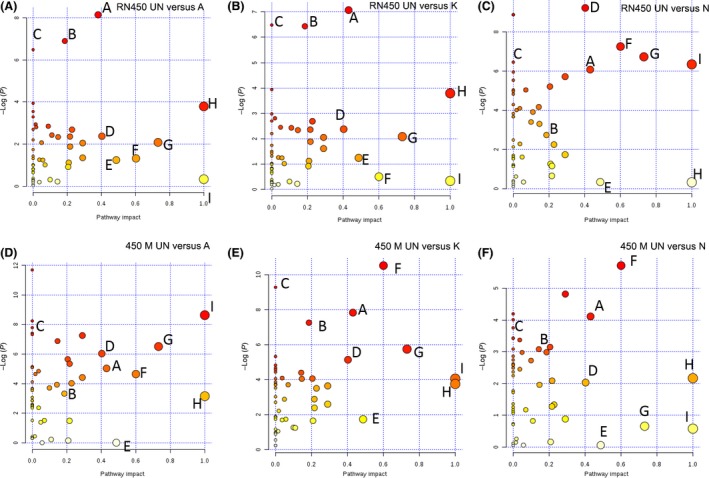
Metabolic pathway analysis revealing metabolic impacts of sublethal dose of antibiotic treatment to *S. aureus* via pairwise comparison. Untreated control groups were compared to either ampicillin, kanamycin or norfloxacin‐treated groups. Top panels, left to right, *S. aureus* RN450 strain dosed with sub‐MIC of ampicillin (RN450‐A, panel A), kanamycin (RN450‐K, panel B) and norfloxacin (RN450‐N, panel C) in comparison with untreated control group (RN450‐UN). Bottom panel, left to right, *S. aureus* 450M strain dosed with sub‐MIC of ampicillin (450M‐A, panel D), kanamycin (450M‐K, panel E) and norfloxacin (450M‐N, panel F) in comparison with untreated control group (450M‐UN). (A) Pyrimidine metabolism; (B) purine metabolism; (C) ascorbate and aldarate metabolism; (D) arginine and proline metabolism; (E) one carbon pool by folate; (F) glycine, serine and threonine metabolism; (G) alanine, aspartate and glutamate metabolism; (H) β‐Alanine metabolism; (I) D‐Alanine metabolism. Dot size corresponding to the pathway impact score and dot colour corresponding to the −log(*P*) value. Detailed pairwise pathway impact can be found in Tables S3–S8.

Next, metabolic pathway impact analyses were conducted in a pairwise fashion for the investigation of detailed pathway perturbations from different antibiotics, with each antibiotic treatment compared directly to the untreated group in each strain. As shown in Fig. 6, each dot represents a unique metabolic pathway, with the dot size corresponding to the pathway impact score and dot colour corresponding to the −log(*P*) value. The same letter represents the same metabolic pathways across the six pairwise comparisons. In the MSSA RN450 experiments, metabolites from pyrimidine metabolism, purine metabolism and ascorbate and aldarate metabolism were significantly dysregulated by ampicillin and kanamycin treatment, while only medium‐to‐low significance was observed for these metabolic pathways in norfloxacin treatment. In the MRSA 450M experiments, metabolites from pyrimidine and glycine, serine and threonine metabolism were showing significant difference in kanamycin and norfloxacin‐treated group in comparison with the untreated group, while in the case of ampicillin treatment, metabolites from ascorbate and aldarate metabolism and d‐alanine metabolism displayed significant difference comparing to untreated group. More *P*‐value and impact scores from detailed pairwise pathway analyses can be seen in Tables S3–S8.

## Discussion

It is well known that β‐lactams, aminoglycosides and quinolones have their specific targets when used for bacteria treatment, and they act upon different perturbation strategies for bacteria growth and survival, such as binding to targeted proteins for inhibition of elongation and translocation steps, inhibition of cell wall synthesis and disruption of RNA synthesis (Kohanski *et al*., 2010). However, how the bacteria respond to the antibiotics at the biochemical pathway level is not well defined. Further understanding of the disrupted biochemical pathways from antibiotic‐disturbed bacterial cells may help us to discover new insights into the mechanisms of antibiotic action and their consequential resistance development. Therefore, we hypothesized that through studying bacteria metabolic activities postantibiotic treatment we can make some interesting connections and comparisons to the metabolic aspect of their similar and unique response to the sublethal dose of antibiotics.

In this pilot study, we explored the possibility of characterizing a large set of metabolites from MSSA and MRSA strains and comparing their levels after treatment with a sublethal dose of antibiotics from three different classes. Our newly developed targeted metabolic profiling approach enabled the broader coverage of metabolite detection with excellent sensitivity and specificity. The data strongly supported that significant perturbation of bacterial metabolism can be detected after the treatment of antibiotics to bacterial culture, which is consistent with several other published studies (Belenky *et al*., 2015; Peng *et al*., 2015). Metabolites from TCA cycle, NAD metabolism, amino acid metabolism and nucleotide metabolism were reported to have significant changes in these studies. In addition to these metabolites, we also detected changes in metabolites such as glutamine, NAD, NADP and cyclic GMP. Dysregulated metabolic pathways, such as pyrimidine metabolism and purine metabolism, have been previously observed in other antibiotic‐induced metabolic alteration studies from different bacteria (Belenky *et al*., 2015), and they were also detected in this study. In another similar study by Dörries *et al*. (2014), using comprehensive global metabolic profiling of *S. aureus* HG001, a MSSA strain is investigated and ampicillin‐induced metabolic alterations were also reported. They discovered 72 intracellular metabolites with significantly altered after 120 min of ampicillin treatment, although some of these metabolites remain unidentified. In comparison, we confidently detected and identified 34 and 64 dysregulated metabolites after ampicillin treatment in MSSA and MRSA strains respectively. Particularly, they have discovered that many intermediates of the central carbon metabolism (such as succinate, pyruvate and fructose‐6‐p), some amino acids (e.g. alanine), together with several nucleotides were significantly dysregulated, which is partially matched to our study findings.

The uniqueness of this study is that the metabolic profiles from a pair of isogenic methicillin‐susceptible and methicillin‐resistant *S. aureus* were tested and compared in parallel, and interesting similarities and differences of metabolic changes were detected across different strains. In both *S. aureus* strains, ampicillin displayed the strongest perturbation effect on metabolic profiles as a variety of metabolites, ranging from pyrimidine nucleotides, purine nucleotides, amines to amino acids were detected at significantly lower level after ampicillin treatment, in comparison with the untreated control group. PCA analysis also proved that the separation of ampicillin‐treated groups and the untreated control groups were achieved in both MSSA and MRSA strains. By contrast, the kanamycin‐treated MSSA strain displayed a subtle difference in terms of metabolic profile in comparison with untreated group, with semi‐separated metabolic profiles in PCA analysis score plot, and norfloxacin‐treated MRSA strains displayed even more overlapped metabolic profiles in comparison with the untreated MRSA group. These three tested antibiotics‐ampicillin, kanamycin and norfloxacin represented three different classes of antibiotics (β‐lactams, aminoglycosides and quinolones), and the bactericidal mechanisms of these three classes were different. The model of action for β‐lactams is by inhibiting the synthesis of peptidoglycan layer of bacterial cell walls and therefore destroy their cell wall structural integrity (Dwyer *et al*., 2015). Aminoglycosides act by binding to the cytosolic, membrane‐associated bacterial ribosome and inhibit the protein synthesis (Davies and Davies, 2010). While quinolones' mechanism of action is to inhibit DNA gyrase and thereby inhibit bacterial cell division (Yim *et al*., 2006; Blair *et al*., 2015). Therefore, there could be a fundamental difference when they impact the bacterial metabolism, which is reflected by our data from this study. The PCA analysis loading plots, which indicated the contribution of each metabolite to the group separation, are shown in Figs S1 and S2. Taken together, the metabolic profiles from different antibiotic perturbation may be used for interpretation and prediction of the antibiotic model of actions, as recently described by Hoerr *et al*. (2016). Furthermore, the different metabolic response from MSSA and MRSA strains to the same antibiotics raised the potential for the further exploration of metabolically differentiate MSSA and MRSA strains postsublethal dose of antibiotic treatment, which is consistent with our recent findings (Schelli *et al*., 2016).

In summary, this pilot study utilized the state‐of‐art mass spectrometry‐based targeted metabolic profiling technique as a powerful tool for the detection and characterization of a variety of metabolites from antibiotic‐treated *S. aureus* strains. The comparative metabolomics analyses demonstrated that three different classes of antibiotics, β‐lactams, aminoglycosides and quinolones, can induce a massive metabolic shift of tested MSSA and MRSA strains at different levels. The metabolic profile shift is robust and significant for the ampicillin treatment groups in both strains and displayed a case by case effect for the perturbation caused by kanamycin and norfloxacin in both strains. Important metabolic pathways such as pyrimidine metabolism, amino acid metabolism and purine metabolism were among the most significantly altered metabolic pathways. While interesting, we envision that follow‐up studies on a detailed illustration of these metabolic changes in a temporal and dose‐dependent manner could be further investigated in the future to obtain deeper insights into the role metabolism plays in bacterial responses to antibiotic treatment. We hope this work, together with other pioneering progress in the field (Aros‐Calt *et al*., 2015; Belenky *et al*., 2015; Peng *et al*., 2015), will move forward the understanding of antibiotic treatment strategies to *S. aureus* infection and eventually assist future discovery of better therapeutic approaches.

## Experimental procedures

### Bacterial strains and growth condition

Isogenic strains of MSSA (*S. aureus* RN450) and MRSA (*S. aureus* 450M) were used in this study (courtesy of Dr. Gordon L. Archer, Virginia Commonwealth University and Dr. Jane E. Hill, Dartmouth College). These two isogenic strains were chosen because they are closely related and share almost identical genetic background except the antibiotic‐resistant genes (staphylococcal cassette chromosome mecA). Both strains were first grown in tryptic soy broth (BD Diagnostics, Sparks Glencoe, MD, USA) and then M9 minimum medium as recommended by the manufacturer. Bacteria were cultured aerobically for 16 h at 37°C with shaking at 180 rpm, then the cultures were either added with sterilized water (the same water that used to dissolve the antibiotics) or mixed with equal volume of antibiotic to achieve desired final concentration (half minimum inhibitory concentration). The cultures were then incubated under the same condition for another 6 h before final optical density (OD) measurement and metabolite extraction. Three biological replicates for each group were prepared for the study.

### Methicillin minimum inhibitory concentration (MIC) test

Ampicillin, kanamycin and norfloxacin were purchased from Fisher Scientific (Pittsburgh, PA, USA). The minimum inhibitory concentration of the two studied strains was determined by a 96‐well plate method as described previously (Wiegand *et al*., 2008). After serial dilution of bacterial culture, ~1 × 10^5^ cfu ml^−1^ of bacteria were inoculated into wells containing different concentrations of ampicillin, kanamycin or norfloxacin solution. Then the 96‐well plates were incubated at 37°C for 16 h with shaking at 180 rpm. Ten μl of culture from each well was then plated on a tryptic soy agar plate to count the number of survived bacterial colonies. The tests were run in triplicate and the average of the plate‐counting results was used for determination of MIC.

### Metabolite extraction

Intracellular metabolites from each biological replicate were extracted using a cold methanol extraction approach as previously reported (Zhu *et al*., 2014, 2015; Schelli *et al*., 2016; Xu *et al*., 2017). Briefly, after co‐incubation with or without antibiotics, one ml of bacterial culture from each biological sample was transferred into a 2 ml Eppendorf tube. The bacterial cells were harvested by centrifugation at 14 000 rpm for 10 min, followed by phosphate buffer saline (PBS) washes. Two hundred and fifty microlitre methanol was added to the cell pellet and the samples were mixed vigorously for ~1 min on a vortex machine, then 50 μl isotope‐labelled amino acid mixture was added as internal standards (Cambridge Isotope Laboratories, Tewksbury, MA, USA) and then mixed one more time. The mixture was stored at −20°C for 20 min and centrifuged for 20 min. Then 150 μl of the supernatant was collected and dried on a vacuum concentrator. The dried sample was later reconstituted by 50% H_2_O and 50% acetonitrile and kept in 4°C auto sampler for MS runs.

### Targeted metabolic profiling

A Thermo Scientific Ultimate 3000 HPLC coupled with a TSQ Quantiva Triple Quadrupole mass spectrometer was used in this study. A hydrophilic interaction chromatography (HILIC) column was purchased from Waters Corporation (Milford, MA, USA). The chromatography method operated as previously described (Zhu *et al*., 2014, 2015). Briefly, analytical grade standard chemical compounds corresponding to the measured metabolites were purchased from Sigma‐Aldrich (Saint Louis, MO, USA) or IROA Technologies (Boston, MA, USA). LC–MS‐grade acetonitrile, ammonium acetate and acetic acid were all purchased from Fisher Scientific. We used flow rate of 0.300 ml min^−1^ for HPLC separation, with autosampler temperature kept at 4°C, and the column compartment set at 40°C. The total separation for both positive and negative ionization modes was 20 min. The mobile phase was composed of 5 mM ammonium acetate in 90% H_2_O/10% acetonitrile + 0.2% acetic acid as solvent A and 5 mM ammonium acetate in 10% H_2_O/90% acetonitrile + 0.2% acetic acid as solvent B. The gradient separation lasts for 11 min and then the column was washed for additional 9 min to avoid potential carryover. The targeted metabolic profiling was performed in selected‐reaction‐monitoring (SRM) mode, established by running multiple standards first, and then using the obtained retention time and SRM transition information to search for the presence of targeted metabolites from unknown samples. Detailed metabolic pathways investigated and metabolites detected have been previously reported and validated (Schelli *et al*., 2016; Xu *et al*., 2017; Zhong *et al*., 2017). Pooled quality control (QC) samples were also run between every ten samples to ensure the performance of the method.

### Data processing and statistical analyses

Mass spectrometry data analysis work flow was similar to our previously published work (Schelli *et al*., 2016; Xu *et al*., 2017). Briefly, all raw data were manually inspected using the Quan browser module of XCALIBUR version 4.0 (Thermo Fisher Scientific). The mass spectrometry data were exported into an Excel spreadsheet and normalized by the bacterial OD measurement at the point of metabolite extraction. Then the data were filtered using the threshold of 80% (meaning the metabolite must be detectable in 80% of the samples from the same group). Data were then processed through log transfer and autoscaling to achieve normal distribution. The reproducibility of our method was defined by the average coefficient of variation value (average CV), which was calculated using standard deviation of each metabolite from all the QC samples divided by their average (defined as coefficient of variation), and then this value is added together and again divided by number of metabolites detected. Both univariate (ANOVA) and multivariate (principal components analysis) statistical analyses were applied using JMP PRO12 (SAS Institute, Cary, NC, USA) and SPSS Version 22.0 (IBM Corp. Armonk, NY, USA). These two methods were selected because they were commonly applied in metabolomics field in understanding both individual metabolite significance from different antibiotic treatment, and the overall metabolic significant that is mostly suitable by multivariate data analysis. Full data set were used for all the principal component analysis. Metabolic pathway analysis was performed using the online tool METABOANALYST 3.0 (http://www.metaboanalyst.ca/; Xia *et al*., 2015).

## Conflict of interest

None declared.

## Supporting information


**Fig. S1.** Principal component analysis (PCA) loading plot showing the contribution of each individual metabolite to the distinctive metabolic profiles between *S. aureus* under different antibiotic treatments. (A). *S. aureus* RN450 strain dosed with sub‐MIC of ampicillin (RN450‐A), kanamycin (RN450‐K) and norfloxacin (RN450‐N) in comparison to untreated control group (RN450‐UN). (B). *S. aureus* 450 M strain dosed with sub‐MIC of ampicillin (450 M‐A), kanamycin (450 M ‐K) and norfloxacin (450 M ‐N) in comparison to untreated control group (450 M ‐UN). The detailed loading factor for each metabolite can be seen in Table S9 and S10..
**Fig. S2.** Principal component analysis (PCA) loading plot showing the individual contribution from a single metabolite for group separation when comparing metabolic profiles between methicillin susceptible and resistant *S. aureus* (MSSA RN450 and MRSA 450M) from untreated control groups and groups treated with different antibiotics. A. Untreated controls; B. Ampicillin treated groups; C. Kanamycin treated groups; D. Norfloxacin treated groups. The detailed loading factor for each metabolite can be seen in Table S11 to S14.
**Table S1.** Analysis of variance (ANOVA) identify significantly (*P* < 0.05) changed metabolites in comparison of the three antibiotics dosed groups and the untreated control in RN450 experiments.
**Table S2.** Analysis of variance (ANOVA) identify significantly (*P* < 0.05) changed metabolites in comparison of the three antibiotics dosed groups and the untreated control in 450M experiments.
**Table S3.** Metabolic pathway impact analysis revealing the significantly impacted metabolic pathways in comparison of untreated RN450 group and RN450 treated with ampicillin.
**Table S4.** Metabolic pathway impact analysis revealing the significantly impacted metabolic pathways in comparison of untreated RN450 group and RN450 treated with kanamycin.
**Table S5.** Metabolic pathway impact analysis revealing the significantly impacted metabolic pathways in comparison of untreated RN450 group and RN450 treated with norfloxacin.
**Table S6.** Metabolic pathway impact analysis revealing the significantly impacted metabolic pathways in comparison of untreated 450M group and RN450 treated with ampicillin.
**Table S7.** Metabolic pathway impact analysis revealing the significantly impacted metabolic pathways in comparison of untreated 450M group and RN450 treated with kanamycin.
**Table S8.** Metabolic pathway impact analysis revealing the significantly impacted metabolic pathways in comparison of untreated 450M group and RN450 treated with norfloxacin.
**Table S9.** The loading factors for each individual metabolite from Figure S1‐A.
**Table S10.** The loading factors for each individual metabolite from Figure S1‐B.
**Table S11.** The loading factors for each individual metabolite from Figure S2‐A.
**Table S12.** The loading factors for each individual metabolite from Figure S2‐B.
**Table S13.** The loading factors for each individual metabolite from Figure S2‐C.
**Table S14.** The loading factors for each individual metabolite from Figure S2‐D.
