# Supplementary material for: Comparative metabolomics revealing Staphylococcus aureus metabolic response to different antibiotics
Source: Microb Biotechnol. 2017 Aug 16;10(6):1764–74. doi: 10.1111/1751-7915.12839 (PMC5658637; doi:10.1111/1751-7915.12839)
Supplement: Supplementary file 1 — Fig. S1. Principal component analysis (PCA) loading plot showing the contribution of each individual metabolite to the distinctive metabolic profiles between S. aureus under different antibiotic treatments. (A). S. aureus RN450 strain dosed with sub‐MIC of ampicillin (RN450‐A), kanamycin (RN450‐K) and norfloxacin (RN450‐N) in comparison to untreated control group (RN450‐UN). (B). S. aureus 450 M strain dosed with sub‐MIC of ampicillin (450 M‐A), kanamycin (450 M ‐K) and norfloxacin (450 M ‐N) in comparison to untreated control group (450 M ‐UN). The detailed loading factor for each metabolite can be seen in Table S9 and S10.. Fig. S2. Principal component analysis (PCA) loading plot showing the individual contribution from a single metabolite for group separation when comparing metabolic profiles between methicillin susceptible and resistant S. aureus (MSSA RN450 and MRSA 450M) from untreated control groups and groups treated with different antibiotics. A. Untreated controls; B. Ampicillin treated groups; C. Kanamycin treated groups; D. Norfloxacin treated groups. The detailed loading factor for each metabolite can be seen in Table S11 to S14. Table S1. Analysis of variance (ANOVA) identify significantly (P < 0.05) changed metabolites in comparison of the three antibiotics dosed groups and the untreated control in RN450 experiments. Table S2. Analysis of variance (ANOVA) identify significantly (P < 0.05) changed metabolites in comparison of the three antibiotics dosed groups and the untreated control in 450M experiments. Table S3. Metabolic pathway impact analysis revealing the significantly impacted metabolic pathways in comparison of untreated RN450 group and RN450 treated with ampicillin. Table S4. Metabolic pathway impact analysis revealing the significantly impacted metabolic pathways in comparison of untreated RN450 group and RN450 treated with kanamycin. Table S5. Metabolic pathway impact analysis revealing the significantly impacted metabolic pathways in comp [file MBT2-10-1764-s001.docx]

**Comparative metabolomics revealing *Staphylococcus aureus* metabolic response to different antibiotics**

Katie Schelli^†^, Fanyi Zhong^†^, Jiangjiang Zhu*

Department of Chemistry and Biochemistry, Miami University

651 E High St., Oxford, OH 45056, USA

^†^These two authors contribute equally to this work

*Corresponding author.

Email address: [zhuj6@miamioh.edu](mailto:zhuj6@miamioh.edu);

Tel: +1 513 529 3998;

Fax: +1 513 529 5715

Figure S1. Principal component analysis (PCA) loading plot showing the contribution of each individual metabolite to the distinctive metabolic profiles between *S. aureus* under different antibiotic treatments. (A). *S. aureus* RN450 strain dosed with sub-MIC of ampicillin (RN450-A), kanamycin (RN450-K) and norfloxacin (RN450-N) in comparison to untreated control group (RN450-UN). (B). *S. aureus* 450 M strain dosed with sub-MIC of ampicillin (450 M-A), kanamycin (450 M -K) and norfloxacin (450 M -N) in comparison to untreated control group (450 M -UN). The detailed loading factor for each metabolite can be seen in Table S9 and S10.

Figure S2. Principal component analysis (PCA) loading plot showing the individual contribution from a single metabolite for group separation when comparing metabolic profiles between methicillin susceptible and resistant *S. aureus* (MSSA RN450 and MRSA 450M) from untreated control groups and groups treated with different antibiotics. A. Untreated controls; B. Ampicillin treated groups; C. Kanamycin treated groups; D. Norfloxacin treated groups. The detailed loading factor for each metabolite can be seen in Table S11 to S14.


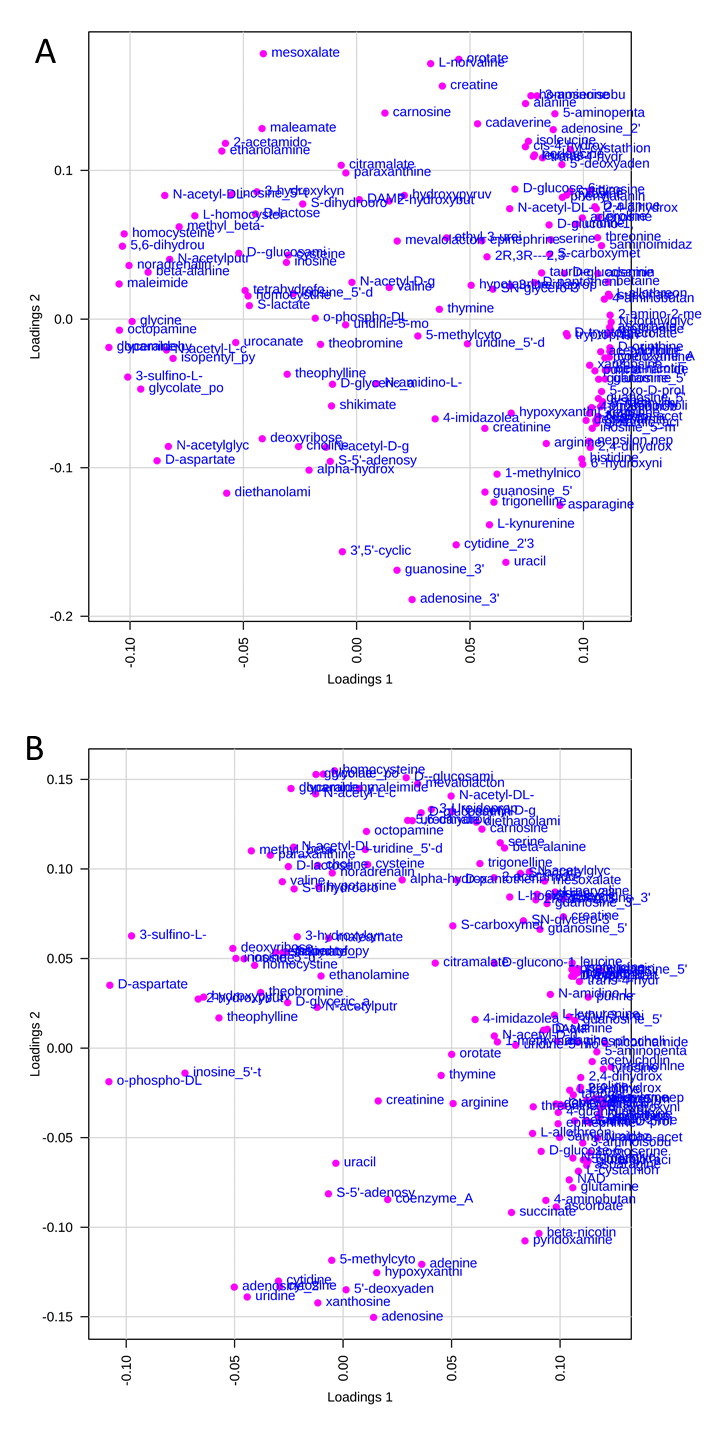


Figure S1


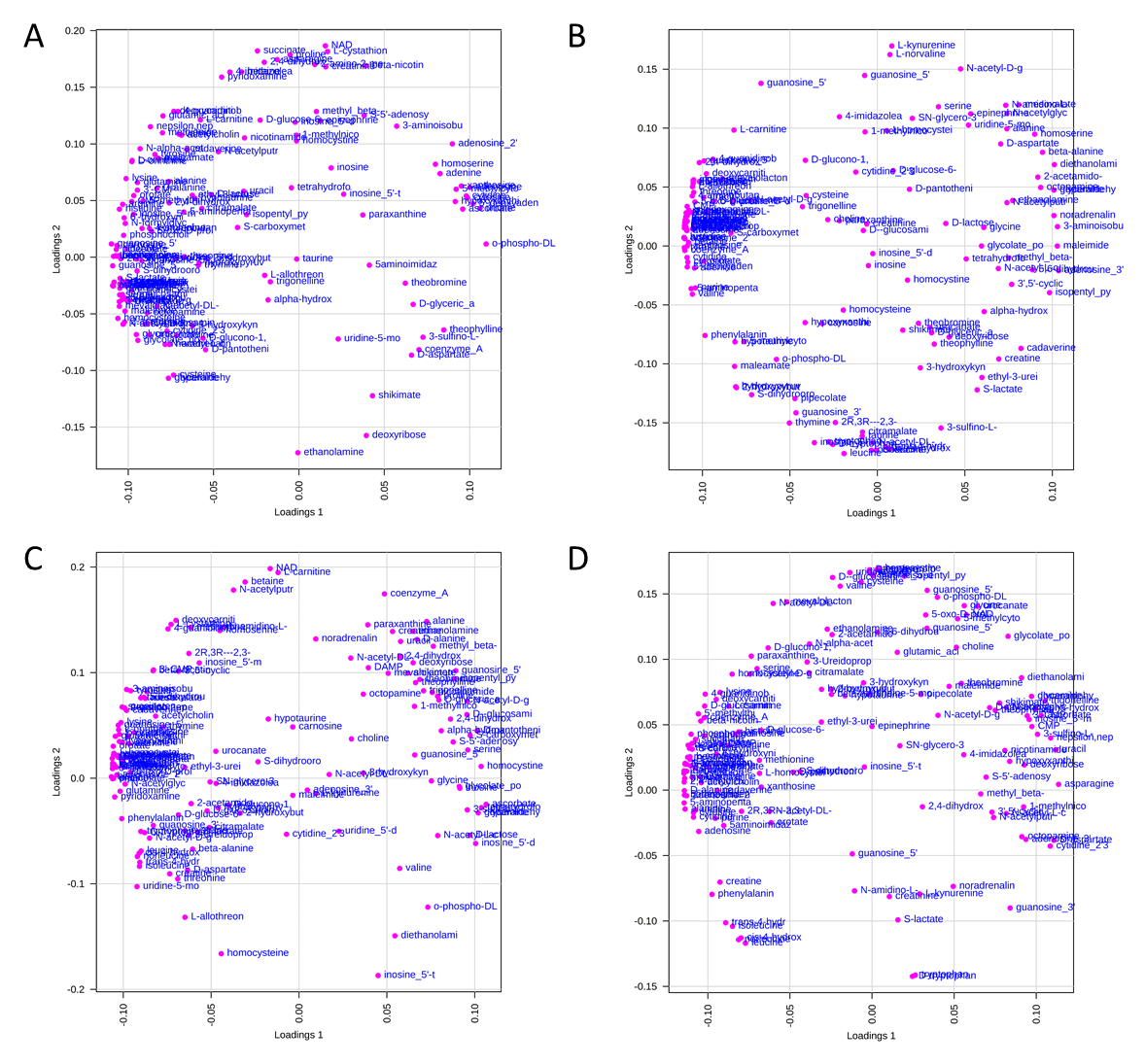


Figure S2

Table S1. Analysis of variance (ANOVA) identify significantly (*p<0.05*) changed metabolites in comparison of the three antibiotics dosed groups and the untreated control in RN450 experiments.

| **Metabolites** | **f.value** | **p.value** | **-LOG p** | **FDR** |
| --- | --- | --- | --- | --- |
| 2-amino-2-methyl propanoate | 686.22 | 5.52E-10 | 9.26 | 8.61E-08 |
| betaine | 411.42 | 4.22E-09 | 8.37 | 3.01E-07 |
| Nε,Nε,Nε-Trimethyllysine | 379.90 | 5.79E-09 | 8.24 | 3.01E-07 |
| AICAR | 342.34 | 8.76E-09 | 8.06 | 3.42E-07 |
| 5-aminopentanoate | 323.03 | 1.10E-08 | 7.96 | 3.44E-07 |
| guanosine 5'-diphosphate | 305.66 | 1.37E-08 | 7.86 | 3.50E-07 |
| ascorbate | 295.46 | 1.57E-08 | 7.80 | 3.50E-07 |
| D-orinthine | 279.47 | 1.96E-08 | 7.71 | 3.67E-07 |
| phosphocholine | 270.35 | 2.23E-08 | 7.65 | 3.67E-07 |
| L-orinthine | 266.69 | 2.35E-08 | 7.63 | 3.67E-07 |
| 5-oxo-D-proline | 245.58 | 3.26E-08 | 7.49 | 4.63E-07 |
| N-formylglycine | 238.19 | 3.68E-08 | 7.43 | 4.79E-07 |
| tyrosine | 205.26 | 6.62E-08 | 7.18 | 7.95E-07 |
| glutamic acid | 199.47 | 7.41E-08 | 7.13 | 8.26E-07 |
| pipecolate | 191.81 | 8.65E-08 | 7.06 | 8.50E-07 |
| 2,4-dihydroxypyrimidine-5-carboxylic acid | 191.42 | 8.72E-08 | 7.06 | 8.50E-07 |
| homoserine | 184.37 | 1.01E-07 | 7.00 | 9.28E-07 |
| 5'-methylthioadenosine | 160.56 | 1.74E-07 | 6.76 | 1.51E-06 |
| L-cystathionine | 152.16 | 2.15E-07 | 6.67 | 1.65E-06 |
| N-alpha-acetyl-L-lysine | 152.13 | 2.15E-07 | 6.67 | 1.65E-06 |
| histidine | 151.03 | 2.21E-07 | 6.65 | 1.65E-06 |
| asparagine | 146.93 | 2.47E-07 | 6.61 | 1.74E-06 |
| lysine | 144.06 | 2.67E-07 | 6.57 | 1.74E-06 |
| proline | 143.93 | 2.67E-07 | 6.57 | 1.74E-06 |
| 2,4-dihydroxypteridine | 131.84 | 3.77E-07 | 6.42 | 2.35E-06 |
| D-alanine | 127.01 | 4.36E-07 | 6.36 | 2.62E-06 |
| methionine | 123.83 | 4.82E-07 | 6.32 | 2.78E-06 |
| 6'-hydroxynicotinate | 114.42 | 6.56E-07 | 6.18 | 3.66E-06 |
| L-allothreonine | 111.22 | 7.33E-07 | 6.14 | 3.94E-06 |
| inosine 5'-monophosphate | 106.02 | 8.83E-07 | 6.05 | 4.59E-06 |
| coenzyme A | 92.30 | 1.51E-06 | 5.82 | 7.61E-06 |
| threonine | 89.77 | 1.68E-06 | 5.77 | 7.83E-06 |
| xanthosine | 89.59 | 1.70E-06 | 5.77 | 7.83E-06 |
| succinate | 89.47 | 1.71E-06 | 5.77 | 7.83E-06 |
| NAD | 81.16 | 2.49E-06 | 5.60 | 1.11E-05 |
| beta-nicotinamideadeninedinucleotidephosphate | 77.64 | 2.95E-06 | 5.53 | 1.28E-05 |
| glutamine | 74.29 | 3.50E-06 | 5.46 | 1.44E-05 |
| 5'-deoxyadenosine | 74.24 | 3.51E-06 | 5.46 | 1.44E-05 |
| CMP | 70.40 | 4.30E-06 | 5.37 | 1.72E-05 |
| 3-aminoisobutanoate | 69.32 | 4.56E-06 | 5.34 | 1.78E-05 |
| purine | 68.05 | 4.90E-06 | 5.31 | 1.87E-05 |
| adenosine 2',3'-cyclic monophosphate | 64.82 | 5.90E-06 | 5.23 | 2.19E-05 |
| cytidine | 64.47 | 6.03E-06 | 5.22 | 2.19E-05 |
| 4-aminobutanoate | 60.73 | 7.58E-06 | 5.12 | 2.69E-05 |
| cytosine | 53.30 | 1.24E-05 | 4.91 | 4.15E-05 |
| glyceraldehyde | 53.24 | 1.25E-05 | 4.90 | 4.15E-05 |
| dopamine | 53.24 | 1.25E-05 | 4.90 | 4.15E-05 |
| guanosine 5'-triphosphate | 52.38 | 1.33E-05 | 4.88 | 4.32E-05 |
| 3'-CMP | 48.51 | 1.78E-05 | 4.75 | 5.65E-05 |
| nicotinamide | 47.50 | 1.92E-05 | 4.72 | 6.00E-05 |
| uridine | 45.95 | 2.18E-05 | 4.66 | 6.66E-05 |
| adenosine | 38.96 | 4.04E-05 | 4.39 | 1.21E-04 |
| alanine | 33.03 | 7.43E-05 | 4.13 | 2.19E-04 |
| mesoxalate | 30.29 | 1.02E-04 | 3.99 | 2.95E-04 |
| pyridoxamine | 26.32 | 1.70E-04 | 3.77 | 4.81E-04 |
| D-aspartate | 25.55 | 1.89E-04 | 3.72 | 5.26E-04 |
| homocysteine | 24.65 | 2.15E-04 | 3.67 | 5.87E-04 |
| adenine | 20.49 | 4.12E-04 | 3.39 | 1.11E-03 |
| octopamine | 19.35 | 5.03E-04 | 3.30 | 1.33E-03 |
| uracil | 18.84 | 5.52E-04 | 3.26 | 1.43E-03 |
| 5,6-dihydrouracil | 18.03 | 6.42E-04 | 3.19 | 1.64E-03 |
| acetylcholine | 17.79 | 6.73E-04 | 3.17 | 1.69E-03 |
| maleimide | 17.67 | 6.88E-04 | 3.16 | 1.70E-03 |
| trigonelline | 17.19 | 7.56E-04 | 3.12 | 1.84E-03 |
| 3-sulfino-L-alanine | 16.45 | 8.77E-04 | 3.06 | 2.11E-03 |
| D-glucosamine 6-sulfate | 15.43 | 1.09E-03 | 2.96 | 2.57E-03 |
| adenosine 3',5'-cyclic monophosphate | 15.04 | 1.19E-03 | 2.93 | 2.77E-03 |
| orotate | 13.17 | 1.84E-03 | 2.74 | 4.22E-03 |
| 4-guanidinobutanoate | 12.93 | 1.95E-03 | 2.71 | 4.42E-03 |
| L-carnitine | 12.56 | 2.15E-03 | 2.67 | 4.78E-03 |
| noradrenaline | 11.91 | 2.55E-03 | 2.59 | 5.60E-03 |
| glycolate positive mode | 11.49 | 2.85E-03 | 2.54 | 6.18E-03 |
| deoxycarnitine | 10.93 | 3.34E-03 | 2.48 | 7.11E-03 |
| L-norvaline | 10.90 | 3.37E-03 | 2.47 | 7.11E-03 |
| guanosine 3',5'-cyclic monophosphate | 9.34 | 5.43E-03 | 2.26 | 1.12E-02 |
| glycine | 9.33 | 5.45E-03 | 2.26 | 1.12E-02 |
| beta-alanine | 9.19 | 5.71E-03 | 2.24 | 1.16E-02 |
| phenylalanine | 9.12 | 5.84E-03 | 2.23 | 1.17E-02 |
| DAMP | 8.32 | 7.67E-03 | 2.12 | 1.52E-02 |
| tryptophan | 7.58 | 1.00E-02 | 2.00 | 1.96E-02 |
| D-tryptophan | 7.21 | 1.16E-02 | 1.94 | 2.21E-02 |
| trans-4-hydroxyproline | 7.20 | 1.16E-02 | 1.93 | 2.21E-02 |
| taurine | 7.03 | 1.24E-02 | 1.91 | 2.33E-02 |
| N-acetylglycine | 6.82 | 1.35E-02 | 1.87 | 2.51E-02 |
| uridine-5-monophosphate | 6.69 | 1.42E-02 | 1.85 | 2.61E-02 |
| arginine | 6.57 | 1.50E-02 | 1.82 | 2.72E-02 |
| N-acetyl-DL-serine | 6.31 | 1.67E-02 | 1.78 | 3.00E-02 |
| isoleucine | 6.28 | 1.69E-02 | 1.77 | 3.00E-02 |
| norleucine | 5.98 | 1.93E-02 | 1.71 | 3.39E-02 |
| serine | 5.87 | 2.03E-02 | 1.69 | 3.52E-02 |
| 5-methylcytosine hydrochloride | 5.74 | 2.15E-02 | 1.67 | 3.69E-02 |
| leucine | 5.71 | 2.19E-02 | 1.66 | 3.71E-02 |
| L-kynurenine | 5.33 | 2.60E-02 | 1.58 | 4.37E-02 |
| maleamate | 5.29 | 2.66E-02 | 1.58 | 4.39E-02 |
| cis-4-hydroxy-D-proline | 5.28 | 2.67E-02 | 1.57 | 4.39E-02 |
| S-carboxymethyl-L-cysteine | 5.21 | 2.76E-02 | 1.56 | 4.44E-02 |
| D-pantothenic acid | 5.20 | 2.78E-02 | 1.56 | 4.44E-02 |
| methyl beta-D-galactoside | 5.19 | 2.79E-02 | 1.55 | 4.44E-02 |
| 2R,3R---2,3-butanediol | 4.81 | 3.36E-02 | 1.47 | 5.30E-02 |
| SN-glycero-3-phosphocholine | 4.76 | 3.45E-02 | 1.46 | 5.38E-02 |
| epinephrine | 4.74 | 3.48E-02 | 1.46 | 5.38E-02 |
| diethanolamine | 4.67 | 3.61E-02 | 1.44 | 5.52E-02 |
| ethanolamine | 4.65 | 3.65E-02 | 1.44 | 5.52E-02 |
| 3',5'-cyclic AMP | 4.64 | 3.68E-02 | 1.43 | 5.52E-02 |
| N-acetylputrescine | 4.55 | 3.85E-02 | 1.41 | 5.71E-02 |
| guanosine 5'-diphosphoglucose | 4.43 | 4.11E-02 | 1.39 | 6.05E-02 |
| D-glucono-1,5-lactone | 4.28 | 4.45E-02 | 1.35 | 6.48E-02 |
| paraxanthine | 4.21 | 4.61E-02 | 1.34 | 6.66E-02 |
| cytidine 2'3'-cyclicmonophosphate | 4.11 | 4.89E-02 | 1.31 | 6.99E-02 |

Table S2. Analysis of variance (ANOVA) identify significantly (*p<0.05*) changed metabolites in comparison of the three antibiotics dosed groups and the untreated control in 450 M experiments.

| **Metabolite** | **f.value** | **p.value** | **-LOG p** | **FDR** |
| --- | --- | --- | --- | --- |
| ascorbate | 275.22 | 2.08E-08 | 7.68 | 2.90E-06 |
| pipecolate | 233.17 | 4.00E-08 | 7.40 | 2.90E-06 |
| 2-amino-2-methyl propanoate | 200.58 | 7.25E-08 | 7.14 | 2.90E-06 |
| AICAR | 199.37 | 7.43E-08 | 7.13 | 2.90E-06 |
| proline | 142.58 | 2.78E-07 | 6.56 | 8.66E-06 |
| 3-aminoisobutanoate | 128.09 | 4.22E-07 | 6.37 | 9.45E-06 |
| N-alpha-acetyl-L-lysine | 127.97 | 4.24E-07 | 6.37 | 9.45E-06 |
| 2,4-dihydroxypyrimidine-5-carboxylic acid | 114.93 | 6.45E-07 | 6.19 | 1.26E-05 |
| glutamic acid | 90.04 | 1.66E-06 | 5.78 | 2.89E-05 |
| D-alanine | 65.19 | 5.78E-06 | 5.24 | 8.71E-05 |
| homoserine | 62.72 | 6.70E-06 | 5.17 | 8.71E-05 |
| 5-oxo-D-proline | 62.71 | 6.70E-06 | 5.17 | 8.71E-05 |
| methionine | 60.01 | 7.93E-06 | 5.10 | 9.51E-05 |
| phosphocholine | 58.72 | 8.61E-06 | 5.06 | 9.60E-05 |
| glutamine | 56.05 | 1.03E-05 | 4.99 | 1.07E-04 |
| L-cystathionine | 55.13 | 1.09E-05 | 4.96 | 1.07E-04 |
| N-formylglycine | 52.45 | 1.32E-05 | 4.88 | 1.21E-04 |
| asparagine | 49.94 | 1.59E-05 | 4.80 | 1.38E-04 |
| histidine | 47.46 | 1.93E-05 | 4.71 | 1.58E-04 |
| 5'-methylthioadenosine | 45.22 | 2.31E-05 | 4.64 | 1.80E-04 |
| alanine | 44.37 | 2.48E-05 | 4.60 | 1.85E-04 |
| pyridoxamine | 42.15 | 3.01E-05 | 4.52 | 2.14E-04 |
| CMP | 34.22 | 6.52E-05 | 4.19 | 4.42E-04 |
| 3'-CMP | 32.51 | 7.88E-05 | 4.10 | 5.12E-04 |
| lysine | 31.20 | 9.15E-05 | 4.04 | 5.71E-04 |
| 6'-hydroxynicotinate | 30.14 | 1.04E-04 | 3.98 | 6.23E-04 |
| inosine 5'-monophosphate | 29.45 | 1.13E-04 | 3.95 | 6.53E-04 |
| beta-nicotinamideadeninedinucleotidephosphate | 28.94 | 1.20E-04 | 3.92 | 6.71E-04 |
| L-homocysteine thiolactone | 28.55 | 1.26E-04 | 3.90 | 6.80E-04 |
| uridine | 26.68 | 1.62E-04 | 3.79 | 8.40E-04 |
| 2,4-dihydroxypteridine | 26.06 | 1.76E-04 | 3.75 | 8.85E-04 |
| L-orinthine | 25.02 | 2.03E-04 | 3.69 | 9.49E-04 |
| tyrosine | 24.94 | 2.06E-04 | 3.69 | 9.49E-04 |
| L-allothreonine | 24.91 | 2.07E-04 | 3.68 | 9.49E-04 |
| D-orinthine | 24.46 | 2.21E-04 | 3.66 | 9.50E-04 |
| Nε,Nε,Nε-Trimethyllysine | 24.43 | 2.22E-04 | 3.65 | 9.50E-04 |
| 5,6-dihydrouracil | 24.31 | 2.25E-04 | 3.65 | 9.50E-04 |
| S-carboxymethyl-L-cysteine | 22.94 | 2.77E-04 | 3.56 | 1.14E-03 |
| NAD | 21.54 | 3.46E-04 | 3.46 | 1.38E-03 |
| betaine | 21.21 | 3.65E-04 | 3.44 | 1.42E-03 |
| threonine | 18.41 | 5.98E-04 | 3.22 | 2.28E-03 |
| adenosine | 18.08 | 6.37E-04 | 3.20 | 2.37E-03 |
| cadaverine | 17.85 | 6.65E-04 | 3.18 | 2.37E-03 |
| mesoxalate | 17.82 | 6.69E-04 | 3.17 | 2.37E-03 |
| guanosine 5'-diphosphate | 17.55 | 7.05E-04 | 3.15 | 2.39E-03 |
| L-norvaline | 17.55 | 7.05E-04 | 3.15 | 2.39E-03 |
| 4-aminobutanoate | 17.35 | 7.32E-04 | 3.14 | 2.43E-03 |
| succinate | 17.17 | 7.59E-04 | 3.12 | 2.47E-03 |
| adenosine 3',5'-cyclic monophosphate | 14.10 | 1.47E-03 | 2.83 | 4.66E-03 |
| adenosine 2',3'-cyclic monophosphate | 13.97 | 1.52E-03 | 2.82 | 4.66E-03 |
| 5-aminopentanoate | 13.95 | 1.52E-03 | 2.82 | 4.66E-03 |
| cytidine | 13.12 | 1.86E-03 | 2.73 | 5.59E-03 |
| xanthosine | 13.02 | 1.91E-03 | 2.72 | 5.62E-03 |
| 5-methylcytosine hydrochloride | 12.19 | 2.36E-03 | 2.63 | 6.71E-03 |
| hypoxyxanthine | 12.19 | 2.36E-03 | 2.63 | 6.71E-03 |
| taurine | 11.90 | 2.56E-03 | 2.59 | 7.12E-03 |
| homocysteine | 11.59 | 2.78E-03 | 2.56 | 7.60E-03 |
| orotate | 11.51 | 2.84E-03 | 2.55 | 7.63E-03 |
| cytosine | 11.39 | 2.94E-03 | 2.53 | 7.77E-03 |
| arginine | 10.32 | 4.00E-03 | 2.40 | 1.04E-02 |
| nicotinamide | 10.24 | 4.10E-03 | 2.39 | 1.05E-02 |
| serine | 10.13 | 4.24E-03 | 2.37 | 1.07E-02 |
| D-aspartate | 9.88 | 4.58E-03 | 2.34 | 1.13E-02 |
| N-acetylglycine | 9.59 | 5.00E-03 | 2.30 | 1.22E-02 |
| guanosine 3',5'-cyclic monophosphate | 9.51 | 5.14E-03 | 2.29 | 1.23E-02 |
| uracil | 9.43 | 5.28E-03 | 2.28 | 1.25E-02 |
| 3-Ureidopropionate | 9.33 | 5.45E-03 | 2.26 | 1.27E-02 |
| purine | 9.26 | 5.57E-03 | 2.25 | 1.28E-02 |
| 3',5'-cyclic AMP | 8.99 | 6.09E-03 | 2.22 | 1.38E-02 |
| creatine | 8.57 | 7.02E-03 | 2.15 | 1.57E-02 |
| methyl beta-D-galactoside | 8.20 | 7.99E-03 | 2.10 | 1.75E-02 |
| isoleucine | 8.15 | 8.15E-03 | 2.09 | 1.77E-02 |
| cis-4-hydroxy-D-proline | 7.71 | 9.55E-03 | 2.02 | 2.02E-02 |
| guanosine 5'-diphosphoglucose | 7.68 | 9.66E-03 | 2.02 | 2.02E-02 |
| tryptophan | 7.61 | 9.93E-03 | 2.00 | 2.02E-02 |
| trans-4-hydroxyproline | 7.60 | 9.96E-03 | 2.00 | 2.02E-02 |
| 5'-deoxyadenosine | 7.60 | 9.98E-03 | 2.00 | 2.02E-02 |
| D-glucose-6-phosphate | 7.55 | 1.02E-02 | 1.99 | 2.03E-02 |
| paraxanthine | 7.43 | 1.06E-02 | 1.97 | 2.05E-02 |
| acetylcholine | 7.42 | 1.07E-02 | 1.97 | 2.05E-02 |
| L-carnitine | 7.41 | 1.07E-02 | 1.97 | 2.05E-02 |
| norleucine | 7.39 | 1.08E-02 | 1.97 | 2.05E-02 |
| D-tryptophan | 7.34 | 1.10E-02 | 1.96 | 2.07E-02 |
| leucine | 7.26 | 1.13E-02 | 1.95 | 2.11E-02 |
| 3-sulfino-L-alanine | 6.71 | 1.42E-02 | 1.85 | 2.60E-02 |
| maleimide | 6.48 | 1.56E-02 | 1.81 | 2.80E-02 |
| D--glucosamine | 6.47 | 1.56E-02 | 1.81 | 2.80E-02 |
| glycolate positive mode | 6.40 | 1.61E-02 | 1.79 | 2.86E-02 |
| glycine | 6.15 | 1.79E-02 | 1.75 | 3.09E-02 |
| L-kynurenine | 6.15 | 1.79E-02 | 1.75 | 3.09E-02 |
| carnosine | 6.13 | 1.81E-02 | 1.74 | 3.09E-02 |
| 4-guanidinobutanoate | 6.11 | 1.82E-02 | 1.74 | 3.09E-02 |
| o-phospho-DL-serine | 5.74 | 2.15E-02 | 1.67 | 3.61E-02 |
| N-acetyl-D-glucosamine | 5.63 | 2.27E-02 | 1.64 | 3.76E-02 |
| beta-alanine | 5.56 | 2.34E-02 | 1.63 | 3.84E-02 |
| epinephrine | 5.44 | 2.48E-02 | 1.61 | 4.02E-02 |
| phenylalanine | 5.37 | 2.55E-02 | 1.59 | 4.10E-02 |
| cytidine 2'3'-cyclicmonophosphate | 5.08 | 2.93E-02 | 1.53 | 4.66E-02 |
| adenine | 5.07 | 2.95E-02 | 1.53 | 4.66E-02 |
| glyceraldehyde | 4.97 | 3.10E-02 | 1.51 | 4.78E-02 |
| dopamine | 4.97 | 3.10E-02 | 1.51 | 4.78E-02 |
| N-acetyl-DL-gluatmic acid | 4.89 | 3.23E-02 | 1.49 | 4.94E-02 |
| SN-glycero-3-phosphocholine | 4.87 | 3.26E-02 | 1.49 | 4.94E-02 |
| mevalolactone | 4.68 | 3.59E-02 | 1.44 | 5.39E-02 |
| diethanolamine | 4.66 | 3.63E-02 | 1.44 | 5.39E-02 |
| S-lactate | 4.40 | 4.16E-02 | 1.38 | 6.12E-02 |
| D-pantothenic acid | 4.18 | 4.68E-02 | 1.33 | 6.83E-02 |

Table S3. Metabolic pathway impact analysis revealing the significantly impacted metabolic pathways in comparison of untreated RN450 group and RN450 treated with ampicillin.

| **Metabolic pathways** | **Total Cmpd^a^** | **Hits^b^** | **-LOG(p)^c^** | **FDR^d^** | **Impact^e^** |
| --- | --- | --- | --- | --- | --- |
| Pyrimidine metabolism | 33 | 10 | 8.14 | 0.015 | 0.38 |
| Purine metabolism | 53 | 13 | 6.90 | 0.026 | 0.19 |
| Ascorbate and aldarate metabolism | 4 | 1 | 6.48 | 0.027 | 0.00 |
| Riboflavin metabolism | 11 | 1 | 3.94 | 0.235 | 0.00 |
| beta-Alanine metabolism | 5 | 2 | 3.79 | 0.235 | 1.00 |
| Lysine degradation | 12 | 2 | 3.54 | 0.250 | 0.00 |
| Glyoxylate and dicarboxylate metabolism | 15 | 3 | 3.29 | 0.276 | 0.00 |
| Glycerolipid metabolism | 15 | 2 | 2.94 | 0.296 | 0.01 |
| Glycolysis or Gluconeogenesis | 29 | 2 | 2.85 | 0.296 | 0.09 |
| Peptidoglycan biosynthesis | 23 | 2 | 2.80 | 0.296 | 0.02 |
| D-Arginine and D-ornithine metabolism | 4 | 1 | 2.70 | 0.296 | 0.00 |
| Glutathione metabolism | 13 | 4 | 2.69 | 0.296 | 0.23 |
| Fatty acid metabolism | 29 | 1 | 2.43 | 0.314 | 0.11 |
| Arginine and proline metabolism | 30 | 8 | 2.37 | 0.314 | 0.40 |
| Pantothenate and CoA biosynthesis | 21 | 5 | 2.36 | 0.314 | 0.22 |
| Nitrogen metabolism | 14 | 5 | 2.34 | 0.314 | 0.15 |
| D-Glutamine and D-glutamate metabolism | 5 | 1 | 2.20 | 0.335 | 0.00 |
| Alanine, aspartate and glutamate metabolism | 18 | 5 | 2.08 | 0.335 | 0.73 |
| Glycerophospholipid metabolism | 17 | 4 | 2.07 | 0.335 | 0.05 |
| Nicotinate and nicotinamide metabolism | 9 | 3 | 2.05 | 0.335 | 0.29 |
| Histidine metabolism | 19 | 4 | 1.88 | 0.377 | 0.22 |
| Lysine biosynthesis | 14 | 2 | 1.74 | 0.417 | 0.00 |
| Cyanoamino acid metabolism | 8 | 3 | 1.42 | 0.518 | 0.00 |
| Cysteine and methionine metabolism | 25 | 6 | 1.35 | 0.518 | 0.29 |
| Glycine, serine and threonine metabolism | 26 | 11 | 1.32 | 0.518 | 0.60 |
| Taurine and hypotaurine metabolism | 7 | 2 | 1.31 | 0.518 | 0.00 |
| Methane metabolism | 13 | 2 | 1.26 | 0.518 | 0.04 |
| One carbon pool by folate | 7 | 1 | 1.24 | 0.518 | 0.49 |
| Folate biosynthesis | 15 | 1 | 1.24 | 0.518 | 0.06 |
| Terpenoid backbone biosynthesis | 18 | 1 | 1.12 | 0.566 | 0.21 |
| Tryptophan metabolism | 14 | 1 | 1.02 | 0.569 | 0.00 |
| Citrate cycle (TCA cycle) | 20 | 1 | 1.01 | 0.569 | 0.07 |
| Benzoate degradation via CoA ligation | 11 | 1 | 1.01 | 0.569 | 0.00 |
| Butanoate metabolism | 16 | 2 | 0.99 | 0.569 | 0.00 |
| Aminoacyl-tRNA biosynthesis | 66 | 19 | 0.92 | 0.595 | 0.21 |
| Pyruvate metabolism | 20 | 1 | 0.82 | 0.596 | 0.00 |
| Tyrosine metabolism | 8 | 1 | 0.82 | 0.596 | 0.00 |
| Novobiocin biosynthesis | 3 | 1 | 0.82 | 0.596 | 0.00 |
| Thiamine metabolism | 13 | 1 | 0.81 | 0.596 | 0.00 |
| Selenoamino acid metabolism | 13 | 1 | 0.70 | 0.648 | 0.00 |
| Propanoate metabolism | 15 | 3 | 0.64 | 0.672 | 0.00 |
| Phenylalanine metabolism | 3 | 1 | 0.38 | 0.846 | 0.00 |
| D-Alanine metabolism | 5 | 2 | 0.33 | 0.846 | 1.00 |
| Phenylalanine, tyrosine and tryptophan biosynthesis | 22 | 4 | 0.31 | 0.846 | 0.10 |
| Galactose metabolism | 21 | 1 | 0.30 | 0.846 | 0.00 |
| Starch and sucrose metabolism | 13 | 1 | 0.27 | 0.846 | 0.00 |
| Streptomycin biosynthesis | 4 | 1 | 0.27 | 0.846 | 0.00 |
| Sulfur metabolism | 11 | 3 | 0.22 | 0.867 | 0.14 |
| Valine, leucine and isoleucine biosynthesis | 26 | 4 | 0.19 | 0.867 | 0.04 |
| Valine, leucine and isoleucine degradation | 25 | 3 | 0.18 | 0.867 | 0.00 |
| Amino sugar and nucleotide sugar metabolism | 28 | 1 | 0.09 | 0.934 | 0.00 |
| Pentose phosphate pathway | 19 | 1 | 0.02 | 0.977 | 0.00 |

Note:

^a^ Total number of metabolites in the pathway

^b^ Number of matched metabolites, explained in Data Analysis section

^c^ -log(P) is the negative natural log of the P value for each pathway shown in Figure 5

^d^ False Discovery Rate (Benjamini-Hochberg)

^e^ Impact is the pathway impact value on each antibiotic treatment calculated from pathway topology analysis

Table S4. Metabolic pathway impact analysis revealing the significantly impacted metabolic pathways in comparison of untreated RN450 group and RN450 treated with kanamycin.

| **Metabolic pathways** | **Total Cmpd^a^** | **Hits^b^** | **-LOG(p)^c^** | **FDR^d^** | **Impact^e^** |
| --- | --- | --- | --- | --- | --- |
| Pyrimidine metabolism | 33 | 10 | 7.07 | 0.028 | 0.43 |
| Ascorbate and aldarate metabolism | 4 | 1 | 6.48 | 0.028 | 0.00 |
| Purine metabolism | 53 | 12 | 6.44 | 0.028 | 0.19 |
| Riboflavin metabolism | 11 | 1 | 3.94 | 0.235 | 0.00 |
| beta-Alanine metabolism | 5 | 2 | 3.79 | 0.235 | 1.00 |
| Amino sugar and nucleotide sugar metabolism | 28 | 2 | 2.98 | 0.359 | 0.00 |
| Peptidoglycan biosynthesis | 23 | 2 | 2.80 | 0.359 | 0.02 |
| D-Arginine and D-ornithine metabolism | 4 | 1 | 2.70 | 0.359 | 0.00 |
| Glutathione metabolism | 13 | 4 | 2.69 | 0.359 | 0.23 |
| Glycerophospholipid metabolism | 17 | 3 | 2.45 | 0.359 | 0.05 |
| Fatty acid metabolism | 29 | 1 | 2.43 | 0.359 | 0.11 |
| Arginine and proline metabolism | 30 | 8 | 2.37 | 0.359 | 0.40 |
| Pantothenate and CoA biosynthesis | 21 | 5 | 2.36 | 0.359 | 0.22 |
| Nitrogen metabolism | 14 | 5 | 2.34 | 0.359 | 0.15 |
| D-Glutamine and D-glutamate metabolism | 5 | 1 | 2.20 | 0.384 | 0.00 |
| Alanine, aspartate and glutamate metabolism | 18 | 5 | 2.08 | 0.394 | 0.73 |
| Nicotinate and nicotinamide metabolism | 9 | 3 | 2.05 | 0.394 | 0.29 |
| Histidine metabolism | 19 | 4 | 1.88 | 0.440 | 0.22 |
| Lysine biosynthesis | 14 | 2 | 1.74 | 0.482 | 0.00 |
| Cysteine and methionine metabolism | 25 | 7 | 1.61 | 0.520 | 0.29 |
| Glyoxylate and dicarboxylate metabolism | 15 | 2 | 1.50 | 0.554 | 0.00 |
| Cyanoamino acid metabolism | 8 | 3 | 1.42 | 0.569 | 0.00 |
| Taurine and hypotaurine metabolism | 7 | 2 | 1.31 | 0.578 | 0.00 |
| Methane metabolism | 13 | 2 | 1.26 | 0.578 | 0.04 |
| One carbon pool by folate | 7 | 1 | 1.24 | 0.578 | 0.49 |
| Folate biosynthesis | 15 | 1 | 1.24 | 0.578 | 0.06 |
| Terpenoid backbone biosynthesis | 18 | 1 | 1.12 | 0.611 | 0.21 |
| Tryptophan metabolism | 14 | 1 | 1.02 | 0.611 | 0.00 |
| Citrate cycle (TCA cycle) | 20 | 1 | 1.01 | 0.611 | 0.07 |
| Benzoate degradation via CoA ligation | 11 | 1 | 1.01 | 0.611 | 0.00 |
| Butanoate metabolism | 16 | 2 | 0.99 | 0.611 | 0.00 |
| Aminoacyl-tRNA biosynthesis | 66 | 19 | 0.92 | 0.611 | 0.21 |
| Lysine degradation | 12 | 2 | 0.84 | 0.611 | 0.00 |
| Glycolysis or Gluconeogenesis | 29 | 1 | 0.82 | 0.611 | 0.00 |
| Pyruvate metabolism | 20 | 1 | 0.82 | 0.611 | 0.00 |
| Tyrosine metabolism | 8 | 1 | 0.82 | 0.611 | 0.00 |
| Novobiocin biosynthesis | 3 | 1 | 0.82 | 0.611 | 0.00 |
| Thiamine metabolism | 13 | 1 | 0.81 | 0.611 | 0.00 |
| Selenoamino acid metabolism | 13 | 1 | 0.70 | 0.664 | 0.00 |
| Propanoate metabolism | 15 | 3 | 0.64 | 0.688 | 0.00 |
| Glycine, serine and threonine metabolism | 26 | 10 | 0.50 | 0.770 | 0.60 |
| Phenylalanine metabolism | 3 | 1 | 0.38 | 0.846 | 0.00 |
| D-Alanine metabolism | 5 | 2 | 0.33 | 0.846 | 1.00 |
| Phenylalanine, tyrosine and tryptophan biosynthesis | 22 | 4 | 0.31 | 0.846 | 0.10 |
| Galactose metabolism | 21 | 1 | 0.30 | 0.846 | 0.00 |
| Starch and sucrose metabolism | 13 | 1 | 0.27 | 0.846 | 0.00 |
| Streptomycin biosynthesis | 4 | 1 | 0.27 | 0.846 | 0.00 |
| Sulfur metabolism | 11 | 3 | 0.22 | 0.858 | 0.14 |
| Valine, leucine and isoleucine biosynthesis | 26 | 4 | 0.19 | 0.858 | 0.04 |
| Valine, leucine and isoleucine degradation | 25 | 3 | 0.18 | 0.858 | 0.00 |
| Glycerolipid metabolism | 15 | 1 | 0.17 | 0.858 | 0.01 |
| Pentose phosphate pathway | 19 | 1 | 0.02 | 0.977 | 0.00 |

Note:

^a^ Total number of metabolites in the pathway

^b^ Number of matched metabolites, explained in Data Analysis section

^c^ -log(P) is the negative natural log of the P value for each pathway shown in Figure 5

^d^ False Discovery Rate (Benjamini-Hochberg)

^e^ Impact is the pathway impact value on each antibiotic treatment calculated from pathway topology analysis

Table S5. Metabolic pathway impact analysis revealing the significantly impacted metabolic pathways in comparison of untreated RN450 group and RN450 treated with norfloxacin.

| **Metabolic pathways** | **Total Cmpd^a^** | **Hits^b^** | **-LOG(p)^c^** | **FDR^d^** | **Impact^e^** |
| --- | --- | --- | --- | --- | --- |
| Arginine and proline metabolism | 30 | 8 | 9.22 | 0.002 | 0.40 |
| Tyrosine metabolism | 8 | 1 | 8.87 | 0.002 | 0.00 |
| Novobiocin biosynthesis | 3 | 1 | 8.87 | 0.002 | 0.00 |
| Glycine, serine and threonine metabolism | 26 | 10 | 7.25 | 0.009 | 0.60 |
| Alanine, aspartate and glutamate metabolism | 18 | 5 | 6.73 | 0.012 | 0.73 |
| Ascorbate and aldarate metabolism | 4 | 1 | 6.45 | 0.013 | 0.00 |
| D-Alanine metabolism | 5 | 2 | 6.34 | 0.013 | 1.00 |
| Pyrimidine metabolism | 33 | 10 | 6.08 | 0.015 | 0.43 |
| Cyanoamino acid metabolism | 8 | 3 | 5.95 | 0.015 | 0.00 |
| Cysteine and methionine metabolism | 25 | 7 | 5.72 | 0.017 | 0.29 |
| Selenoamino acid metabolism | 13 | 1 | 5.53 | 0.019 | 0.00 |
| Aminoacyl-tRNA biosynthesis | 66 | 19 | 5.21 | 0.024 | 0.21 |
| Citrate cycle (TCA cycle) | 20 | 1 | 5.04 | 0.024 | 0.07 |
| Benzoate degradation via CoA ligation | 11 | 1 | 5.04 | 0.024 | 0.00 |
| Butanoate metabolism | 16 | 2 | 4.97 | 0.024 | 0.00 |
| D-Arginine and D-ornithine metabolism | 4 | 1 | 4.83 | 0.026 | 0.00 |
| Taurine and hypotaurine metabolism | 7 | 2 | 4.29 | 0.042 | 0.00 |
| Sulfur metabolism | 11 | 3 | 4.17 | 0.045 | 0.14 |
| Valine, leucine and isoleucine biosynthesis | 26 | 4 | 4.10 | 0.045 | 0.04 |
| Peptidoglycan biosynthesis | 23 | 2 | 4.01 | 0.047 | 0.02 |
| Fatty acid metabolism | 29 | 1 | 3.91 | 0.049 | 0.11 |
| Lysine biosynthesis | 14 | 2 | 3.84 | 0.051 | 0.00 |
| Starch and sucrose metabolism | 13 | 1 | 3.67 | 0.055 | 0.00 |
| Streptomycin biosynthesis | 4 | 1 | 3.67 | 0.055 | 0.00 |
| Phenylalanine, tyrosine and tryptophan biosynthesis | 22 | 4 | 3.41 | 0.069 | 0.10 |
| Nitrogen metabolism | 14 | 5 | 3.31 | 0.073 | 0.15 |
| Purine metabolism | 53 | 12 | 2.74 | 0.124 | 0.19 |
| Phenylalanine metabolism | 3 | 1 | 2.49 | 0.154 | 0.00 |
| Methane metabolism | 13 | 2 | 2.29 | 0.182 | 0.04 |
| Glutathione metabolism | 13 | 4 | 2.26 | 0.182 | 0.23 |
| Valine, leucine and isoleucine degradation | 25 | 3 | 2.14 | 0.198 | 0.00 |
| Nicotinate and nicotinamide metabolism | 9 | 3 | 1.74 | 0.285 | 0.29 |
| Lysine degradation | 12 | 2 | 1.71 | 0.286 | 0.00 |
| Propanoate metabolism | 15 | 3 | 1.62 | 0.294 | 0.00 |
| Glycerophospholipid metabolism | 17 | 3 | 1.62 | 0.294 | 0.05 |
| Pentose phosphate pathway | 19 | 1 | 1.54 | 0.305 | 0.00 |
| Glyoxylate and dicarboxylate metabolism | 15 | 2 | 1.53 | 0.305 | 0.00 |
| Terpenoid backbone biosynthesis | 18 | 1 | 1.27 | 0.384 | 0.21 |
| D-Glutamine and D-glutamate metabolism | 5 | 1 | 1.23 | 0.386 | 0.00 |
| Glycolysis or Gluconeogenesis | 29 | 1 | 1.17 | 0.386 | 0.00 |
| Pyruvate metabolism | 20 | 1 | 1.17 | 0.386 | 0.00 |
| Histidine metabolism | 19 | 4 | 1.17 | 0.386 | 0.22 |
| Amino sugar and nucleotide sugar metabolism | 28 | 2 | 1.14 | 0.388 | 0.00 |
| Pantothenate and CoA biosynthesis | 21 | 5 | 0.66 | 0.610 | 0.22 |
| Glycerolipid metabolism | 15 | 1 | 0.63 | 0.617 | 0.01 |
| Riboflavin metabolism | 11 | 1 | 0.41 | 0.744 | 0.00 |
| One carbon pool by folate | 7 | 1 | 0.35 | 0.744 | 0.49 |
| Folate biosynthesis | 15 | 1 | 0.35 | 0.744 | 0.06 |
| Galactose metabolism | 21 | 1 | 0.34 | 0.744 | 0.00 |
| beta-Alanine metabolism | 5 | 2 | 0.34 | 0.744 | 1.00 |
| Tryptophan metabolism | 14 | 1 | 0.23 | 0.812 | 0.00 |
| Thiamine metabolism | 13 | 1 | 0.12 | 0.890 | 0.00 |

Note:

^a^ Total number of metabolites in the pathway

^b^ Number of matched metabolites, explained in Data Analysis section

^c^ -log(P) is the negative natural log of the P value for each pathway shown in Figure 5

^d^ False Discovery Rate (Benjamini-Hochberg)

^e^ Impact is the pathway impact value on each antibiotic treatment calculated from pathway topology analysis

Table S6. Metabolic pathway impact analysis revealing the significantly impacted metabolic pathways in comparison of untreated 450 M group and RN450 treated with ampicillin.

| **Metabolic pathways** | **Total Cmpd^a^** | **Hits^b^** | **-LOG(p)^c^** | **FDR^d^** | **Impact^e^** |
| --- | --- | --- | --- | --- | --- |
| Taurine and hypotaurine metabolism | 7 | 2 | 11.69 | 4.36E-04 | 0.00 |
| D-Alanine metabolism | 5 | 2 | 8.63 | 4.58E-03 | 1.00 |
| Selenoamino acid metabolism | 13 | 1 | 8.24 | 4.58E-03 | 0.00 |
| Ascorbate and aldarate metabolism | 4 | 1 | 7.78 | 5.26E-03 | 0.00 |
| Lysine biosynthesis | 14 | 2 | 7.41 | 5.26E-03 | 0.00 |
| D-Glutamine and D-glutamate metabolism | 5 | 1 | 7.32 | 5.26E-03 | 0.00 |
| Cysteine and methionine metabolism | 25 | 6 | 7.25 | 5.26E-03 | 0.29 |
| Nitrogen metabolism | 14 | 5 | 6.87 | 6.72E-03 | 0.15 |
| Alanine, aspartate and glutamate metabolism | 18 | 5 | 6.51 | 8.62E-03 | 0.73 |
| Arginine and proline metabolism | 30 | 8 | 6.03 | 1.25E-02 | 0.40 |
| Aminoacyl-tRNA biosynthesis | 66 | 19 | 5.65 | 1.67E-02 | 0.21 |
| Histidine metabolism | 19 | 4 | 5.34 | 2.07E-02 | 0.22 |
| D-Arginine and D-ornithine metabolism | 4 | 1 | 5.15 | 2.13E-02 | 0.00 |
| Tyrosine metabolism | 8 | 1 | 5.09 | 2.13E-02 | 0.00 |
| Novobiocin biosynthesis | 3 | 1 | 5.09 | 2.13E-02 | 0.00 |
| Pyrimidine metabolism | 33 | 10 | 5.03 | 2.13E-02 | 0.43 |
| Valine, leucine and isoleucine biosynthesis | 26 | 4 | 4.84 | 2.42E-02 | 0.04 |
| Peptidoglycan biosynthesis | 23 | 2 | 4.65 | 2.63E-02 | 0.02 |
| Glycine, serine and threonine metabolism | 26 | 10 | 4.64 | 2.63E-02 | 0.60 |
| Nicotinate and nicotinamide metabolism | 9 | 3 | 4.41 | 3.15E-02 | 0.29 |
| Cyanoamino acid metabolism | 8 | 3 | 4.12 | 4.03E-02 | 0.00 |
| Glutathione metabolism | 13 | 4 | 4.02 | 4.24E-02 | 0.23 |
| Sulfur metabolism | 11 | 3 | 3.93 | 4.46E-02 | 0.14 |
| Phenylalanine, tyrosine and tryptophan biosynthesis | 22 | 4 | 3.71 | 5.25E-02 | 0.10 |
| Lysine degradation | 12 | 2 | 3.67 | 5.25E-02 | 0.00 |
| Tryptophan metabolism | 14 | 1 | 3.64 | 5.25E-02 | 0.00 |
| Valine, leucine and isoleucine degradation | 25 | 3 | 3.53 | 5.64E-02 | 0.00 |
| Purine metabolism | 53 | 12 | 3.32 | 6.71E-02 | 0.19 |
| beta-Alanine metabolism | 5 | 2 | 3.15 | 7.68E-02 | 1.00 |
| Phenylalanine metabolism | 3 | 1 | 3.06 | 8.11E-02 | 0.00 |
| Starch and sucrose metabolism | 13 | 1 | 2.85 | 9.42E-02 | 0.00 |
| Streptomycin biosynthesis | 4 | 1 | 2.85 | 9.42E-02 | 0.00 |
| Propanoate metabolism | 15 | 3 | 2.60 | 1.17E-01 | 0.00 |
| Butanoate metabolism | 16 | 2 | 2.55 | 1.18E-01 | 0.00 |
| Glycolysis or Gluconeogenesis | 29 | 1 | 2.51 | 1.18E-01 | 0.00 |
| Pyruvate metabolism | 20 | 1 | 2.51 | 1.18E-01 | 0.00 |
| Amino sugar and nucleotide sugar metabolism | 28 | 2 | 2.41 | 1.26E-01 | 0.00 |
| Methane metabolism | 13 | 2 | 2.36 | 1.27E-01 | 0.04 |
| Riboflavin metabolism | 11 | 1 | 2.35 | 1.27E-01 | 0.00 |
| Citrate cycle (TCA cycle) | 20 | 1 | 1.51 | 2.81E-01 | 0.07 |
| Benzoate degradation via CoA ligation | 11 | 1 | 1.51 | 2.81E-01 | 0.00 |
| Pantothenate and CoA biosynthesis | 21 | 5 | 1.48 | 2.82E-01 | 0.22 |
| Glycerophospholipid metabolism | 17 | 3 | 1.38 | 3.03E-01 | 0.05 |
| Glycerolipid metabolism | 15 | 1 | 0.43 | 7.64E-01 | 0.01 |
| Galactose metabolism | 21 | 1 | 0.41 | 7.64E-01 | 0.00 |
| Pentose phosphate pathway | 19 | 1 | 0.33 | 8.07E-01 | 0.00 |
| Thiamine metabolism | 13 | 1 | 0.30 | 8.07E-01 | 0.00 |
| Glyoxylate and dicarboxylate metabolism | 15 | 2 | 0.29 | 8.07E-01 | 0.00 |
| Fatty acid metabolism | 29 | 1 | 0.22 | 8.54E-01 | 0.11 |
| Terpenoid backbone biosynthesis | 18 | 1 | 0.15 | 8.98E-01 | 0.21 |
| One carbon pool by folate | 7 | 1 | 0.00 | 9.99E-01 | 0.49 |
| Folate biosynthesis | 15 | 1 | 0.00 | 9.99E-01 | 0.06 |

Note:

^a^ Total number of metabolites in the pathway

^b^ Number of matched metabolites, explained in Data Analysis section

^c^ -log(P) is the negative natural log of the P value for each pathway shown in Figure 5

^d^ False Discovery Rate (Benjamini-Hochberg)

^e^ Impact is the pathway impact value on each antibiotic treatment calculated from pathway topology analysis

Table S7. Metabolic pathway impact analysis revealing the significantly impacted metabolic pathways in comparison of untreated 450 M group and RN450 treated with kanamycin.

| **Metabolic pathways** | **Total Cmpd^a^** | **Hits^b^** | **-LOG(p)^c^** | **FDR^d^** | **Impact^e^** |
| --- | --- | --- | --- | --- | --- |
| Glycine, serine and threonine metabolism | 26 | 10 | 10.52 | 1.41E-03 | 0.60 |
| Ascorbate and aldarate metabolism | 4 | 1 | 9.27 | 2.44E-03 | 0.00 |
| Pyrimidine metabolism | 33 | 10 | 7.83 | 6.91E-03 | 0.43 |
| Purine metabolism | 53 | 12 | 7.25 | 9.20E-03 | 0.19 |
| Alanine, aspartate and glutamate metabolism | 18 | 5 | 5.75 | 3.32E-02 | 0.73 |
| Propanoate metabolism | 15 | 3 | 5.33 | 4.21E-02 | 0.00 |
| Arginine and proline metabolism | 30 | 8 | 5.14 | 4.37E-02 | 0.40 |
| Cyanoamino acid metabolism | 8 | 3 | 4.83 | 5.19E-02 | 0.00 |
| Taurine and hypotaurine metabolism | 7 | 2 | 4.67 | 5.30E-02 | 0.00 |
| Butanoate metabolism | 16 | 2 | 4.57 | 5.30E-02 | 0.00 |
| Galactose metabolism | 21 | 1 | 4.44 | 5.30E-02 | 0.00 |
| Sulfur metabolism | 11 | 3 | 4.40 | 5.30E-02 | 0.14 |
| Methane metabolism | 13 | 2 | 4.09 | 5.30E-02 | 0.04 |
| Aminoacyl-tRNA biosynthesis | 66 | 19 | 4.06 | 5.30E-02 | 0.21 |
| Selenoamino acid metabolism | 13 | 1 | 4.06 | 5.30E-02 | 0.00 |
| D-Alanine metabolism | 5 | 2 | 4.06 | 5.30E-02 | 1.00 |
| Nitrogen metabolism | 14 | 5 | 4.05 | 5.30E-02 | 0.15 |
| Amino sugar and nucleotide sugar metabolism | 28 | 2 | 3.87 | 5.30E-02 | 0.00 |
| beta-Alanine metabolism | 5 | 2 | 3.75 | 5.30E-02 | 1.00 |
| D-Glutamine and D-glutamate metabolism | 5 | 1 | 3.73 | 5.30E-02 | 0.00 |
| Starch and sucrose metabolism | 13 | 1 | 3.72 | 5.30E-02 | 0.00 |
| Streptomycin biosynthesis | 4 | 1 | 3.72 | 5.30E-02 | 0.00 |
| Citrate cycle (TCA cycle) | 20 | 1 | 3.71 | 5.30E-02 | 0.07 |
| Benzoate degradation via CoA ligation | 11 | 1 | 3.71 | 5.30E-02 | 0.00 |
| Cysteine and methionine metabolism | 25 | 7 | 3.65 | 5.43E-02 | 0.29 |
| Glyoxylate and dicarboxylate metabolism | 15 | 2 | 3.53 | 5.78E-02 | 0.00 |
| Glutathione metabolism | 13 | 4 | 3.51 | 5.78E-02 | 0.23 |
| Lysine biosynthesis | 14 | 2 | 3.03 | 8.97E-02 | 0.00 |
| Pantothenate and CoA biosynthesis | 21 | 5 | 2.89 | 9.72E-02 | 0.22 |
| Glycerophospholipid metabolism | 17 | 3 | 2.88 | 9.72E-02 | 0.05 |
| Riboflavin metabolism | 11 | 1 | 2.71 | 1.12E-01 | 0.00 |
| Nicotinate and nicotinamide metabolism | 9 | 3 | 2.60 | 1.21E-01 | 0.29 |
| Histidine metabolism | 19 | 4 | 2.39 | 1.44E-01 | 0.22 |
| Peptidoglycan biosynthesis | 23 | 2 | 2.22 | 1.67E-01 | 0.02 |
| Glycolysis or Gluconeogenesis | 29 | 1 | 1.89 | 2.19E-01 | 0.00 |
| Pyruvate metabolism | 20 | 1 | 1.89 | 2.19E-01 | 0.00 |
| Pentose phosphate pathway | 19 | 1 | 1.85 | 2.21E-01 | 0.00 |
| Valine, leucine and isoleucine degradation | 25 | 3 | 1.74 | 2.28E-01 | 0.00 |
| One carbon pool by folate | 7 | 1 | 1.74 | 2.28E-01 | 0.49 |
| Folate biosynthesis | 15 | 1 | 1.74 | 2.28E-01 | 0.06 |
| Valine, leucine and isoleucine biosynthesis | 26 | 4 | 1.70 | 2.32E-01 | 0.04 |
| Terpenoid backbone biosynthesis | 18 | 1 | 1.65 | 2.37E-01 | 0.21 |
| Phenylalanine, tyrosine and tryptophan biosynthesis | 22 | 4 | 1.27 | 3.39E-01 | 0.10 |
| Fatty acid metabolism | 29 | 1 | 1.25 | 3.39E-01 | 0.11 |
| Tryptophan metabolism | 14 | 1 | 1.17 | 3.57E-01 | 0.00 |
| Glycerolipid metabolism | 15 | 1 | 1.05 | 3.94E-01 | 0.01 |
| D-Arginine and D-ornithine metabolism | 4 | 1 | 0.99 | 4.12E-01 | 0.00 |
| Phenylalanine metabolism | 3 | 1 | 0.95 | 4.18E-01 | 0.00 |
| Thiamine metabolism | 13 | 1 | 0.89 | 4.35E-01 | 0.00 |
| Lysine degradation | 12 | 2 | 0.56 | 5.97E-01 | 0.00 |
| Tyrosine metabolism | 8 | 1 | 0.24 | 7.85E-01 | 0.00 |
| Novobiocin biosynthesis | 3 | 1 | 0.24 | 7.85E-01 | 0.00 |

Note:

^a^ Total number of metabolites in the pathway

^b^ Number of matched metabolites, explained in Data Analysis section

^c^ -log(P) is the negative natural log of the P value for each pathway shown in Figure 5

^d^ False Discovery Rate (Benjamini-Hochberg)

^e^ Impact is the pathway impact value on each antibiotic treatment calculated from pathway topology analysis

Table S8. Metabolic pathway impact analysis revealing the significantly impacted metabolic pathways in comparison of untreated 450 M group and RN450 treated with norfloxacin.

| **Metabolic pathways** | **Total Cmpd^a^** | **Hits^b^** | **-LOG(p)^c^** | **FDR^d^** | **Impact^e^** |
| --- | --- | --- | --- | --- | --- |
| Glycine, serine and threonine metabolism | 26 | 10 | 5.71 | 1.72E-01 | 0.60 |
| Cysteine and methionine metabolism | 25 | 7 | 4.82 | 1.89E-01 | 0.29 |
| Riboflavin metabolism | 11 | 1 | 4.19 | 1.89E-01 | 0.00 |
| Pyrimidine metabolism | 33 | 10 | 4.11 | 1.89E-01 | 0.43 |
| Lysine biosynthesis | 14 | 2 | 4.01 | 1.89E-01 | 0.00 |
| Ascorbate and aldarate metabolism | 4 | 1 | 3.75 | 1.89E-01 | 0.00 |
| Tyrosine metabolism | 8 | 1 | 3.43 | 1.89E-01 | 0.00 |
| Novobiocin biosynthesis | 3 | 1 | 3.43 | 1.89E-01 | 0.00 |
| Valine, leucine and isoleucine biosynthesis | 26 | 4 | 3.37 | 1.89E-01 | 0.04 |
| Aminoacyl-tRNA biosynthesis | 66 | 19 | 3.15 | 1.89E-01 | 0.21 |
| Propanoate metabolism | 15 | 3 | 3.09 | 1.89E-01 | 0.00 |
| Sulfur metabolism | 11 | 3 | 3.08 | 1.89E-01 | 0.14 |
| Purine metabolism | 53 | 12 | 2.99 | 1.89E-01 | 0.19 |
| Glycerophospholipid metabolism | 17 | 3 | 2.98 | 1.89E-01 | 0.05 |
| Tryptophan metabolism | 14 | 1 | 2.86 | 1.95E-01 | 0.00 |
| Cyanoamino acid metabolism | 8 | 3 | 2.74 | 1.95E-01 | 0.00 |
| Phenylalanine, tyrosine and tryptophan biosynthesis | 22 | 4 | 2.72 | 1.95E-01 | 0.10 |
| Valine, leucine and isoleucine degradation | 25 | 3 | 2.62 | 1.95E-01 | 0.00 |
| Starch and sucrose metabolism | 13 | 1 | 2.58 | 1.95E-01 | 0.00 |
| Streptomycin biosynthesis | 4 | 1 | 2.58 | 1.95E-01 | 0.00 |
| Glycolysis or Gluconeogenesis | 29 | 1 | 2.49 | 1.95E-01 | 0.00 |
| Pyruvate metabolism | 20 | 1 | 2.49 | 1.95E-01 | 0.00 |
| Methane metabolism | 13 | 2 | 2.45 | 1.95E-01 | 0.04 |
| Amino sugar and nucleotide sugar metabolism | 28 | 2 | 2.37 | 2.03E-01 | 0.00 |
| Phenylalanine metabolism | 3 | 1 | 2.25 | 2.20E-01 | 0.00 |
| beta-Alanine metabolism | 5 | 2 | 2.17 | 2.29E-01 | 1.00 |
| Histidine metabolism | 19 | 4 | 2.09 | 2.39E-01 | 0.22 |
| Arginine and proline metabolism | 30 | 8 | 2.03 | 2.44E-01 | 0.40 |
| Nitrogen metabolism | 14 | 5 | 1.96 | 2.53E-01 | 0.15 |
| D-Arginine and D-ornithine metabolism | 4 | 1 | 1.77 | 2.95E-01 | 0.00 |
| Lysine degradation | 12 | 2 | 1.72 | 3.01E-01 | 0.00 |
| Taurine and hypotaurine metabolism | 7 | 2 | 1.36 | 4.15E-01 | 0.00 |
| Glutathione metabolism | 13 | 4 | 1.33 | 4.15E-01 | 0.23 |
| Pantothenate and CoA biosynthesis | 21 | 5 | 1.28 | 4.24E-01 | 0.22 |
| D-Glutamine and D-glutamate metabolism | 5 | 1 | 1.25 | 4.24E-01 | 0.00 |
| Citrate cycle (TCA cycle) | 20 | 1 | 1.17 | 4.34E-01 | 0.07 |
| Benzoate degradation via CoA ligation | 11 | 1 | 1.17 | 4.34E-01 | 0.00 |
| Butanoate metabolism | 16 | 2 | 1.11 | 4.49E-01 | 0.00 |
| Glyoxylate and dicarboxylate metabolism | 15 | 2 | 1.06 | 4.61E-01 | 0.00 |
| Nicotinate and nicotinamide metabolism | 9 | 3 | 0.88 | 5.37E-01 | 0.29 |
| Fatty acid metabolism | 29 | 1 | 0.82 | 5.57E-01 | 0.11 |
| Thiamine metabolism | 13 | 1 | 0.73 | 5.94E-01 | 0.00 |
| Galactose metabolism | 21 | 1 | 0.71 | 5.95E-01 | 0.00 |
| Alanine, aspartate and glutamate metabolism | 18 | 5 | 0.66 | 6.13E-01 | 0.73 |
| D-Alanine metabolism | 5 | 2 | 0.58 | 6.47E-01 | 1.00 |
| Peptidoglycan biosynthesis | 23 | 2 | 0.26 | 8.72E-01 | 0.02 |
| Terpenoid backbone biosynthesis | 18 | 1 | 0.16 | 9.37E-01 | 0.21 |
| Glycerolipid metabolism | 15 | 1 | 0.14 | 9.37E-01 | 0.01 |
| Pentose phosphate pathway | 19 | 1 | 0.12 | 9.42E-01 | 0.00 |
| One carbon pool by folate | 7 | 1 | 0.06 | 9.51E-01 | 0.49 |
| Folate biosynthesis | 15 | 1 | 0.06 | 9.51E-01 | 0.06 |
| Selenoamino acid metabolism | 13 | 1 | 0.05 | 9.51E-01 | 0.00 |

Note:

^a^ Total number of metabolites in the pathway

^b^ Number of matched metabolites, explained in Data Analysis section

^c^ -log(P) is the negative natural log of the P value for each pathway shown in Figure 5

^d^ False Discovery Rate (Benjamini-Hochberg)

^e^ Impact is the pathway impact value on each antibiotic treatment calculated from pathway topology analysis

Table S9. The loading factors for each individual metabolite from Figure S1-A.

| Metabolites | LOADING 1 | LOADING 2 |
| --- | --- | --- |
| uridine 5'-diphosphate | 0.048875 | -0.01665 |
| deoxyribose | -0.04166 | -0.08054 |
| alpha-hydroxyisobutyric acid | -0.02102 | -0.10166 |
| homocystine | -0.04793 | 0.015548 |
| inosine | -0.03098 | 0.038116 |
| isopentyl pyrophosphate | -0.08102 | -0.02648 |
| D-glucose-6-phosphate | 0.069832 | 0.087415 |
| shikimate | -0.01108 | -0.05844 |
| tetrahydrofolate | -0.04922 | 0.019192 |
| D-glyceric acid | -0.01066 | -0.04388 |
| inosine 5'-diphosphate | -0.02795 | 0.01714 |
| mevalolactone | 0.017892 | 0.052499 |
| D-lactose | -0.04464 | 0.070714 |
| paraxanthine | -0.00477 | 0.098355 |
| theophylline | -0.03065 | -0.03713 |
| 5'-methylthioadenosine | 0.10837 | -0.0573 |
| thymine | 0.036492 | 0.006566 |
| nicotinamide | 0.10969 | -0.03441 |
| theobromine | -0.01595 | -0.01705 |
| 2,4-dihydroxypyrimidine-5-carboxylic acid | 0.10574 | 0.074254 |
| 5'-deoxyadenosine | 0.090563 | 0.10393 |
| D-pantothenic acid | 0.078544 | 0.024423 |
| purine | 0.10513 | -0.03482 |
| 2,4-dihydroxypteridine | 0.10306 | -0.08642 |
| uridine | 0.096969 | 0.063854 |
| urocanate | -0.05337 | -0.01578 |
| D--glucosamine | -0.05202 | 0.044282 |
| adenosine | 0.099562 | 0.068067 |
| creatinine | 0.056561 | -0.07354 |
| 3',5'-cyclic AMP | -0.0063 | -0.15653 |
| D-glucono-1,5-lactone | 0.084906 | 0.063371 |
| 3-Ureidopropionate | 0.067954 | 0.022316 |
| adenosine 3',5'-cyclic monophosphate | 0.024472 | -0.18873 |
| tryptophan | 0.092991 | -0.01128 |
| L-kynurenine | 0.058591 | -0.13841 |
| phenylalanine | 0.090776 | 0.081905 |
| D-tryptophan | 0.092535 | -0.00974 |
| isoleucine | 0.075796 | 0.11962 |
| leucine | 0.078039 | 0.10935 |
| norleucine | 0.078452 | 0.11047 |
| 6'-hydroxynicotinate | 0.099748 | -0.09763 |
| N-acetyl-DL-gluatmic acid | 0.067526 | 0.074253 |
| 5-oxo-D-proline | 0.10807 | -0.04876 |
| acetylcholine | 0.10768 | -0.022 |
| N-acetyl-L-cysteine | -0.08389 | -0.02081 |
| cytidine | 0.094183 | 0.085758 |
| cytidine 2'3'-cyclicmonophosphate | 0.043899 | -0.1519 |
| methionine | 0.11207 | -0.00734 |
| N-acetyl-D-glucosamine | -0.01355 | -0.08627 |
| 3-hydroxykynurenine | -0.04404 | 0.08561 |
| N-acetyl-D-galactosamine | -0.00208 | 0.024698 |
| 5-methylcytosine hydrochloride | 0.026999 | -0.01139 |
| uridine-5-monophosphate | -0.00489 | -0.00386 |
| cytosine | 0.092618 | 0.083433 |
| trigonelline | 0.060558 | -0.12326 |
| tyrosine | 0.10303 | 0.086466 |
| citramalate | -0.00682 | 0.10348 |
| betaine | 0.11092 | 0.024935 |
| proline | 0.10671 | 0.06857 |
| taurine | 0.081605 | 0.031058 |
| valine | 0.014376 | 0.021228 |
| pipecolate | 0.11099 | -0.01023 |
| D-aspartate | -0.08807 | -0.09531 |
| maleamate | -0.04177 | 0.12819 |
| 5,6-dihydrouracil | -0.10335 | 0.049022 |
| hypotaurine | 0.050515 | 0.02272 |
| cysteine | -0.03017 | 0.043066 |
| S-carboxymethyl-L-cysteine | 0.085111 | 0.043648 |
| 2R,3R---2,3-butanediol | 0.057522 | 0.041876 |
| glycolate positive mode | -0.09528 | -0.0471 |
| trans-4-hydroxyproline | 0.081995 | 0.1085 |
| cis-4-hydroxy-D-proline | 0.074467 | 0.11596 |
| glycine | -0.09908 | -0.00162 |
| mesoxalate | -0.04118 | 0.17856 |
| 2-amino-2-methyl propanoate | 0.11184 | 0.002637 |
| 3-aminoisobutanoate | 0.079584 | 0.1502 |
| alanine | 0.074511 | 0.14501 |
| diethanolamine | -0.05734 | -0.11716 |
| L-homocysteine thiolactone | -0.0714 | 0.069494 |
| L-norvaline | 0.03264 | 0.17186 |
| N-acetylglycine | -0.08308 | -0.08571 |
| N-amidino-L-aspartate | 0.008802 | -0.04343 |
| ethyl-3-ureidopropionate | 0.039661 | 0.054633 |
| N-acetyl-DL-serine | -0.08467 | 0.083156 |
| 3-sulfino-L-alanine | -0.10101 | -0.03902 |
| glyceraldehyde | -0.10934 | -0.01911 |
| maleimide | -0.10481 | 0.023606 |
| 1-methylnicotinamide | 0.061938 | -0.10435 |
| 4-guanidinobutanoate | 0.10369 | -0.05957 |
| epinephrine | 0.05528 | 0.052686 |
| noradrenaline | -0.10044 | 0.036037 |
| octopamine | -0.10466 | -0.0075 |
| choline | -0.02567 | -0.08576 |
| N-acetylputrescine | -0.08252 | 0.040166 |
| N-formylglycine | 0.1123 | -0.00208 |
| 4-aminobutanoate | 0.10928 | 0.013392 |
| dopamine | -0.10934 | -0.01911 |
| D-alanine | 0.10511 | 0.075639 |
| ethanolamine | -0.05948 | 0.11306 |
| N-alpha-acetyl-L-lysine | 0.10628 | -0.06605 |
| creatine | 0.037813 | 0.1568 |
| 5-aminopentanoate | 0.087461 | 0.13812 |
| homoserine | 0.07691 | 0.15028 |
| L-carnitine | 0.10271 | -0.06342 |
| threonine | 0.10629 | 0.054778 |
| beta-alanine | -0.09203 | 0.031519 |
| L-allothreonine | 0.11112 | 0.016749 |
| SN-glycero-3-phosphocholine | 0.060097 | 0.020113 |
| 4-imidazoleacetic acid | 0.03464 | -0.06717 |
| 5aminoimidazole4carboxamide1betaDribofurano | 0.10805 | 0.049383 |
| S-5'-adenosyl-L-homocysteine | -0.01166 | -0.0958 |
| deoxycarnitine | 0.10122 | -0.06818 |
| hydroxypyruvate | 0.021042 | 0.08321 |
| inosine 5'-monophosphate | 0.10394 | -0.07353 |
| 2-hydroxybutyric acid | 0.014321 | 0.079459 |
| coenzyme A | 0.11148 | -0.0257 |
| glutamine | 0.10964 | -0.04035 |
| inosine 5'-triphosphate | -0.0551 | 0.083905 |
| serine | 0.085695 | 0.053382 |
| asparagine | 0.089703 | -0.12537 |
| D-glucosamine 6-sulfate | 0.091662 | 0.030813 |
| 2-acetamido-2-deoxy-beta-D-glucosylamine | -0.0579 | 0.11823 |
| pyridoxamine | 0.10952 | -0.02611 |
| glutamic acid | 0.10584 | -0.07015 |
| NAD | 0.10662 | -0.06551 |
| nepsilon,nepsilon,nepsilon,-trimethyllysine | 0.10262 | -0.0822 |
| carnosine | 0.012472 | 0.13861 |
| histidine | 0.099295 | -0.09411 |
| homocysteine | -0.10258 | 0.057302 |
| o-phospho-DL-serine | -0.01827 | 0.000589 |
| arginine | 0.083677 | -0.08374 |
| lysine | 0.1085 | -0.05557 |
| D-orinthine | 0.11177 | -0.0193 |
| L-orinthine | 0.1117 | -0.02177 |
| cadaverine | 0.053341 | 0.13141 |
| beta-nicotinamideadeninedinucleotidephosphate | 0.11075 | -0.03385 |
| guanosine 5'-diphosphoglucose | 0.056616 | -0.11642 |
| phosphocholine | 0.10761 | -0.059 |
| methyl beta-D-galactoside | -0.07834 | 0.062069 |
| uracil | 0.065841 | -0.16368 |
| succinate | 0.11162 | 0.015529 |
| hypoxyxanthine | 0.068182 | -0.06327 |
| adenine | 0.10641 | 0.030668 |
| orotate | 0.045075 | 0.17476 |
| S-lactate | -0.04734 | 0.009047 |
| adenosine 2',3'-cyclic monophosphate | 0.086739 | 0.12745 |
| xanthosine | 0.10282 | -0.03108 |
| S-dihydroorotate | -0.02381 | 0.07743 |
| ascorbate | 0.11163 | -0.00552 |
| guanosine 3',5'-cyclic monophosphate | 0.017791 | -0.169 |
| 3'-CMP | 0.10603 | -0.068 |
| DAMP | 0.001147 | 0.080393 |
| CMP | 0.10547 | -0.06822 |
| L-cystathionine | 0.094331 | 0.1142 |
| guanosine 5'-diphosphate | 0.10668 | -0.05349 |
| guanosine 5'-triphosphate | 0.10678 | -0.04047 |

Table S10. The loading factors for each individual metabolite from Figure S1-B.

| Metabolite | LOADING 1 | LOADING 2 |
| --- | --- | --- |
| uridine 5'-diphosphate | 0.01021 | 0.11082 |
| deoxyribose | -0.050815 | 0.055625 |
| alpha-hydroxyisobutyric acid | 0.027223 | 0.093867 |
| homocystine | -0.040772 | 0.046178 |
| inosine | -0.045634 | 0.049822 |
| isopentyl pyrophosphate | -0.025908 | 0.053592 |
| D-glucose-6-phosphate | 0.091268 | -0.05766 |
| shikimate | -0.027972 | 0.053278 |
| tetrahydrofolate | -0.031079 | 0.053411 |
| D-glyceric acid | -0.025589 | 0.025368 |
| inosine 5'-diphosphate | -0.049411 | 0.050051 |
| mevalolactone | 0.034331 | 0.14746 |
| D-lactose | -0.02524 | 0.10133 |
| paraxanthine | -0.033565 | 0.10769 |
| theophylline | -0.057229 | 0.016827 |
| 5'-methylthioadenosine | 0.11072 | -0.06242 |
| thymine | 0.045181 | -0.01531 |
| nicotinamide | 0.12075 | 0.002671 |
| theobromine | -0.037992 | 0.030995 |
| 2,4-dihydroxypyrimidine-5-carboxylic acid | 0.10953 | -0.01643 |
| 5'-deoxyadenosine | 0.0013559 | -0.13492 |
| D-pantothenic acid | 0.052562 | 0.093722 |
| purine | 0.11299 | 0.028451 |
| 2,4-dihydroxypteridine | 0.10907 | -0.02288 |
| uridine | -0.044197 | -0.13894 |
| urocanate | 0.031903 | 0.12695 |
| D--glucosamine | 0.029067 | 0.15095 |
| adenosine | 0.014039 | -0.15034 |
| creatinine | 0.016189 | -0.02951 |
| 3',5'-cyclic AMP | 0.094896 | 0.084503 |
| D-glucono-1,5-lactone | 0.069667 | 0.047306 |
| 3-Ureidopropionate | 0.040706 | 0.13325 |
| adenosine 3',5'-cyclic monophosphate | 0.10152 | 0.083302 |
| tryptophan | 0.1063 | 0.040072 |
| L-kynurenine | 0.097387 | 0.018391 |
| phenylalanine | 0.10544 | 0.043884 |
| D-tryptophan | 0.10538 | 0.040112 |
| isoleucine | 0.10816 | 0.044395 |
| leucine | 0.10556 | 0.047553 |
| norleucine | 0.10684 | 0.04116 |
| 6'-hydroxynicotinate | 0.11865 | -0.03353 |
| N-acetyl-DL-gluatmic acid | 0.049782 | 0.14069 |
| 5-oxo-D-proline | 0.11356 | -0.04147 |
| acetylcholine | 0.1148 | -0.00752 |
| N-acetyl-L-cysteine | -0.012668 | 0.14188 |
| cytidine | -0.029793 | -0.13006 |
| cytidine 2'3'-cyclicmonophosphate | 0.089421 | 0.085858 |
| methionine | 0.12355 | -0.01062 |
| N-acetyl-D-glucosamine | 0.050502 | 0.13203 |
| 3-hydroxykynurenine | -0.021135 | 0.062149 |
| N-acetyl-D-galactosamine | 0.069702 | 0.006758 |
| 5-methylcytosine hydrochloride | -0.0052401 | -0.11847 |
| uridine-5-monophosphate | 0.079518 | 0.001586 |
| cytosine | -0.029204 | -0.13316 |
| trigonelline | 0.06305 | 0.10297 |
| tyrosine | 0.11993 | -0.01172 |
| citramalate | 0.04246 | 0.047441 |
| betaine | 0.10674 | -0.04066 |
| proline | 0.10941 | -0.02189 |
| taurine | 0.1061 | -0.02612 |
| valine | -0.028008 | 0.092859 |
| pipecolate | 0.11796 | -0.03853 |
| D-aspartate | -0.10752 | 0.035066 |
| maleamate | -0.0066681 | 0.061401 |
| 5,6-dihydrouracil | 0.029754 | 0.1271 |
| hypotaurine | -0.011232 | 0.090063 |
| cysteine | 0.0113 | 0.10244 |
| S-carboxymethyl-L-cysteine | 0.050574 | 0.068235 |
| 2R,3R---2,3-butanediol | 0.088815 | 0.082759 |
| glycolate positive mode | -0.012505 | 0.15274 |
| trans-4-hydroxyproline | 0.10893 | 0.0372 |
| cis-4-hydroxy-D-proline | 0.10797 | 0.041897 |
| glycine | -0.0091015 | 0.15293 |
| mesoxalate | 0.0929 | 0.09321 |
| 2-amino-2-methyl propanoate | 0.11178 | -0.04072 |
| 3-aminoisobutanoate | 0.11045 | -0.05297 |
| alanine | 0.098685 | 0.003804 |
| diethanolamine | 0.061439 | 0.12611 |
| L-homocysteine thiolactone | 0.076788 | 0.084321 |
| L-norvaline | 0.09772 | 0.087116 |
| N-acetylglycine | 0.085902 | 0.098422 |
| N-amidino-L-aspartate | 0.095512 | 0.02995 |
| ethyl-3-ureidopropionate | 0.10421 | 0.017425 |
| N-acetyl-DL-serine | -0.022794 | 0.11215 |
| 3-sulfino-L-alanine | -0.097507 | 0.062627 |
| glyceraldehyde | -0.024021 | 0.14489 |
| maleimide | 0.0074956 | 0.14497 |
| 1-methylnicotinamide | 0.071104 | 0.003434 |
| 4-guanidinobutanoate | 0.099143 | -0.03592 |
| epinephrine | 0.099086 | -0.04223 |
| noradrenaline | -0.0050008 | 0.097737 |
| octopamine | 0.010765 | 0.12093 |
| choline | -0.011676 | 0.10201 |
| N-acetylputrescine | -0.011948 | 0.022746 |
| N-formylglycine | 0.1059 | -0.06137 |
| 4-aminobutanoate | 0.093452 | -0.08502 |
| dopamine | -0.024021 | 0.14489 |
| D-alanine | 0.094106 | 0.010487 |
| ethanolamine | -0.010182 | 0.040233 |
| N-alpha-acetyl-L-lysine | 0.11731 | -0.05033 |
| creatine | 0.10152 | 0.073303 |
| 5-aminopentanoate | 0.11694 | -0.00211 |
| homoserine | 0.11365 | -0.05824 |
| L-carnitine | 0.10445 | -0.02352 |
| threonine | 0.087628 | -0.03279 |
| beta-alanine | 0.074391 | 0.11171 |
| L-allothreonine | 0.087316 | -0.04765 |
| SN-glycero-3-phosphocholine | 0.083097 | 0.071028 |
| 4-imidazoleacetic acid | 0.060843 | 0.015914 |
| 5aminoimidazole4carboxamide1betaDribofurano | 0.099748 | -0.04985 |
| S-5'-adenosyl-L-homocysteine | -0.0067674 | -0.08136 |
| deoxycarnitine | 0.098085 | -0.03122 |
| hydroxypyruvate | -0.064307 | 0.02846 |
| inosine 5'-monophosphate | 0.11225 | -0.02907 |
| 2-hydroxybutyric acid | -0.066793 | 0.02736 |
| coenzyme A | 0.020531 | -0.08455 |
| glutamine | 0.10584 | -0.07796 |
| inosine 5'-triphosphate | -0.072876 | -0.01399 |
| serine | 0.072415 | 0.1146 |
| asparagine | 0.11228 | -0.06542 |
| D-glucosamine 6-sulfate | 0.035938 | 0.13135 |
| 2-acetamido-2-deoxy-beta-D-glucosylamine | 0.069562 | 0.095102 |
| pyridoxamine | 0.083816 | -0.10763 |
| glutamic acid | 0.11299 | -0.06299 |
| NAD | 0.10423 | -0.07357 |
| nepsilon,nepsilon,nepsilon,-trimethyllysine | 0.11844 | -0.02827 |
| carnosine | 0.06398 | 0.12223 |
| histidine | 0.12027 | -0.03176 |
| homocysteine | -0.0039296 | 0.1548 |
| o-phospho-DL-serine | -0.10796 | -0.01877 |
| arginine | 0.050698 | -0.03096 |
| lysine | 0.11976 | -0.02914 |
| D-orinthine | 0.11806 | -0.03734 |
| L-orinthine | 0.11806 | -0.03853 |
| cadaverine | 0.1161 | -0.02817 |
| beta-nicotinamideadeninedinucleotidephosphate | 0.090241 | -0.10349 |
| guanosine 5'-diphosphoglucose | 0.10683 | 0.015405 |
| phosphocholine | 0.10816 | 0.003297 |
| methyl beta-D-galactoside | -0.042298 | 0.11008 |
| uracil | -0.0033433 | -0.06429 |
| succinate | 0.077619 | -0.09178 |
| hypoxyxanthine | 0.015504 | -0.12541 |
| adenine | 0.036258 | -0.12073 |
| orotate | 0.050032 | -0.00352 |
| S-lactate | 0.081725 | 0.097486 |
| adenosine 2',3'-cyclic monophosphate | -0.050149 | -0.13342 |
| xanthosine | -0.01165 | -0.14224 |
| S-dihydroorotate | -0.022585 | 0.088863 |
| ascorbate | 0.098245 | -0.08871 |
| guanosine 3',5'-cyclic monophosphate | 0.094004 | 0.080659 |
| 3'-CMP | 0.10176 | -0.03221 |
| DAMP | 0.092373 | 0.009973 |
| CMP | 0.10011 | -0.03152 |
| L-cystathionine | 0.10846 | -0.06875 |
| guanosine 5'-diphosphate | 0.11841 | 0.043303 |
| guanosine 5'-triphosphate | 0.090714 | 0.066282 |

Table S11. The loading factors for each individual metabolite from Figure S2-A.

| **Metabolite** | **LOADING 1** | **LOADING 2** |
| --- | --- | --- |
| uridine 5'-diphosphate | -0.07243 | -0.00068 |
| deoxyribose | 0.039459 | -0.15743 |
| alpha-hydroxyisobutyric acid | -0.01807 | -0.03773 |
| homocystine | -0.00137 | 0.10269 |
| inosine | 0.018855 | 0.078827 |
| isopentyl pyrophosphate | -0.03061 | 0.03754 |
| D-glucose-6-phosphate | -0.0227 | 0.12101 |
| shikimate | 0.043074 | -0.12233 |
| tetrahydrofolate | -0.0044 | 0.061284 |
| D-glyceric acid | 0.066889 | -0.04168 |
| inosine 5'-diphosphate | -0.00223 | 0.11912 |
| mevalolactone | -0.10517 | -0.04335 |
| D-lactose | -0.0513 | 0.055202 |
| paraxanthine | 0.037417 | 0.037266 |
| theophylline | 0.084842 | -0.06428 |
| 5'-methylthioadenosine | -0.09137 | 0.049636 |
| thymine | -0.05891 | -0.0072 |
| nicotinamide | -0.03185 | 0.10529 |
| theobromine | 0.062286 | -0.02296 |
| 2,4-dihydroxypyrimidine-5-carboxylic acid | -0.02031 | 0.1721 |
| 5'-deoxyadenosine | 0.10193 | 0.047238 |
| D-pantothenic acid | -0.05466 | -0.08168 |
| purine | -0.10722 | 0.001347 |
| 2,4-dihydroxypteridine | -0.07596 | 0.047911 |
| uridine | 0.10603 | 0.043114 |
| urocanate | -0.08749 | -0.06859 |
| D--glucosamine | -0.1034 | -0.03296 |
| adenosine | 0.10014 | 0.061554 |
| creatinine | 0.015723 | 0.16804 |
| 3',5'-cyclic AMP | -0.10491 | -0.02354 |
| D-glucono-1,5-lactone | -0.05615 | -0.07162 |
| 3-Ureidopropionate | -0.10519 | -0.03922 |
| adenosine 3',5'-cyclic monophosphate | -0.10914 | -0.02505 |
| tryptophan | -0.10189 | -0.02346 |
| L-kynurenine | -0.0919 | 0.025326 |
| phenylalanine | -0.10244 | -0.02172 |
| D-tryptophan | -0.1022 | -0.02351 |
| isoleucine | -0.10091 | -0.02557 |
| leucine | -0.09959 | -0.02208 |
| norleucine | -0.10035 | -0.02505 |
| 6'-hydroxynicotinate | -0.10234 | 0.034728 |
| N-acetyl-DL-gluatmic acid | -0.10354 | -0.03739 |
| 5-oxo-D-proline | -0.08631 | 0.022817 |
| acetylcholine | -0.06881 | 0.10772 |
| N-acetyl-L-cysteine | -0.07839 | -0.07694 |
| cytidine | 0.097991 | 0.054009 |
| cytidine 2'3'-cyclicmonophosphate | -0.07724 | -0.06519 |
| methionine | -0.07992 | 0.10979 |
| N-acetyl-D-glucosamine | -0.103 | -0.05919 |
| 3-hydroxykynurenine | -0.06227 | -0.06038 |
| N-acetyl-D-galactosamine | -0.09941 | -0.03849 |
| 5-methylcytosine hydrochloride | 0.092084 | 0.060021 |
| uridine-5-monophosphate | 0.022986 | -0.07219 |
| cytosine | 0.09877 | 0.052356 |
| trigonelline | -0.01671 | -0.02172 |
| tyrosine | -0.08432 | 0.090647 |
| citramalate | -0.05691 | 0.042937 |
| betaine | -0.0336 | 0.16362 |
| proline | -0.00521 | 0.17851 |
| taurine | -0.0013 | -0.00168 |
| valine | -0.07027 | 0.10864 |
| pipecolate | -0.10698 | 0.006802 |
| D-aspartate | 0.065932 | -0.08649 |
| maleamate | -0.08233 | 0.087337 |
| 5,6-dihydrouracil | -0.10224 | -0.03613 |
| hypotaurine | -0.06093 | 0.051971 |
| cysteine | -0.07344 | -0.10406 |
| S-carboxymethyl-L-cysteine | -0.03595 | 0.026237 |
| 2R,3R---2,3-butanediol | -0.10431 | -0.00143 |
| glycolate positive mode | -0.09431 | -0.07342 |
| trans-4-hydroxyproline | -0.10036 | -0.0217 |
| cis-4-hydroxy-D-proline | -0.09995 | -0.02347 |
| glycine | -0.0952 | -0.06829 |
| mesoxalate | -0.10879 | -0.02548 |
| 2-amino-2-methyl propanoate | 0.009291 | 0.17009 |
| 3-aminoisobutanoate | 0.057243 | 0.11567 |
| alanine | -0.07619 | 0.0668 |
| diethanolamine | -0.10519 | 0.001432 |
| L-homocysteine thiolactone | -0.09797 | -0.02917 |
| L-norvaline | -0.10855 | 0.000751 |
| N-acetylglycine | -0.10826 | -0.02198 |
| N-amidino-L-aspartate | -0.09005 | -0.05914 |
| ethyl-3-ureidopropionate | -0.06246 | 0.054678 |
| N-acetyl-DL-serine | -0.08315 | -0.04334 |
| 3-sulfino-L-alanine | 0.072661 | -0.07067 |
| glyceraldehyde | -0.07642 | -0.10684 |
| maleimide | -0.10188 | -0.04759 |
| 1-methylnicotinamide | -0.00142 | 0.10764 |
| 4-guanidinobutanoate | -0.07087 | 0.12869 |
| epinephrine | 0.012449 | 0.12044 |
| noradrenaline | -0.07584 | -0.07702 |
| octopamine | -0.0887 | -0.04915 |
| choline | -0.0718 | -0.0693 |
| N-acetylputrescine | -0.04717 | 0.093121 |
| N-formylglycine | -0.1017 | 0.029815 |
| 4-aminobutanoate | -0.08752 | 0.025375 |
| dopamine | -0.07642 | -0.10684 |
| D-alanine | -0.08313 | 0.060821 |
| ethanolamine | -0.00062 | -0.17262 |
| N-alpha-acetyl-L-lysine | -0.09302 | 0.095733 |
| creatine | -0.10075 | -0.038 |
| 5-aminopentanoate | -0.06743 | 0.039856 |
| homoserine | 0.079928 | 0.082041 |
| L-carnitine | -0.05772 | 0.12117 |
| threonine | -0.06712 | 0.00047 |
| beta-alanine | -0.10665 | 0.000884 |
| L-allothreonine | -0.02008 | -0.01601 |
| SN-glycero-3-phosphocholine | -0.09523 | -0.00162 |
| 4-imidazoleacetic acid | -0.04046 | 0.16342 |
| 5aminoimidazole4carboxamide1betaDribofurano | 0.04125 | -0.00704 |
| S-5'-adenosyl-L-homocysteine | 0.038168 | 0.12527 |
| deoxycarnitine | -0.07326 | 0.12874 |
| hydroxypyruvate | -0.05846 | -0.00525 |
| inosine 5'-monophosphate | -0.09691 | 0.037849 |
| 2-hydroxybutyric acid | -0.0538 | -0.00122 |
| coenzyme A | 0.070315 | -0.0817 |
| glutamine | -0.09454 | 0.065958 |
| inosine 5'-triphosphate | 0.026197 | 0.055606 |
| serine | -0.10498 | -0.03321 |
| asparagine | -0.01278 | 0.17441 |
| D-glucosamine 6-sulfate | -0.08863 | -0.05845 |
| 2-acetamido-2-deoxy-beta-D-glucosylamine | -0.1021 | -0.05719 |
| pyridoxamine | -0.04524 | 0.15902 |
| glutamic acid | -0.07963 | 0.12471 |
| NAD | 0.015476 | 0.18656 |
| nepsilon,nepsilon,nepsilon,-trimethyllysine | -0.08695 | 0.11517 |
| carnosine | -0.10657 | 0.010404 |
| histidine | -0.10513 | 0.04241 |
| homocysteine | -0.10604 | -0.05401 |
| o-phospho-DL-serine | 0.10961 | 0.011632 |
| arginine | -0.10321 | 0.046642 |
| lysine | -0.10122 | 0.069395 |
| D-orinthine | -0.09783 | 0.084405 |
| L-orinthine | -0.09769 | 0.085394 |
| cadaverine | -0.06561 | 0.095081 |
| beta-nicotinamideadeninedinucleotidephosphate | 0.039242 | 0.16893 |
| guanosine 5'-diphosphoglucose | -0.09201 | -0.00308 |
| phosphocholine | -0.10265 | 0.019305 |
| methyl beta-D-galactoside | 0.010215 | 0.12858 |
| uracil | -0.03239 | 0.058192 |
| succinate | -0.02429 | 0.18211 |
| hypoxyxanthine | 0.091546 | 0.049078 |
| adenine | 0.082633 | 0.07379 |
| orotate | -0.09672 | 0.054383 |
| S-lactate | -0.10474 | -0.01868 |
| adenosine 2',3'-cyclic monophosphate | 0.089846 | 0.10005 |
| xanthosine | 0.095119 | 0.06282 |
| S-dihydroorotate | -0.09478 | -0.01212 |
| ascorbate | 0.095931 | 0.042478 |
| guanosine 3',5'-cyclic monophosphate | -0.10487 | -0.02743 |
| 3'-CMP | -0.09462 | 0.059147 |
| DAMP | -0.10072 | 0.007139 |
| CMP | -0.09792 | 0.049121 |
| L-cystathionine | 0.016742 | 0.18151 |
| guanosine 5'-diphosphate | -0.10982 | 0.011691 |
| guanosine 5'-triphosphate | -0.10908 | -0.00763 |

Table S12. The loading factors for each individual metabolite from Figure S2-B.

| **Metabolite** | **LOADING 1** | **LOADING 2** |
| --- | --- | --- |
| uridine 5'-diphosphate | -0.08268 | 0.037458 |
| deoxyribose | 0.041052 | -0.07731 |
| alpha-hydroxyisobutyric acid | 0.061084 | -0.0557 |
| homocystine | 0.017261 | -0.02895 |
| inosine | -0.00508 | -0.01665 |
| isopentyl pyrophosphate | 0.098436 | -0.03962 |
| D-glucose-6-phosphate | 0.009049 | 0.063963 |
| shikimate | 0.014558 | -0.07139 |
| tetrahydrofolate | 0.050803 | -0.01099 |
| D-glyceric acid | 0.03102 | -0.07364 |
| inosine 5'-diphosphate | -0.00259 | -0.00649 |
| mevalolactone | -0.08842 | 0.05714 |
| D-lactose | 0.039156 | 0.019119 |
| paraxanthine | -0.02163 | 0.021428 |
| theophylline | 0.032557 | -0.08322 |
| 5'-methylthioadenosine | -0.10947 | 0.024662 |
| thymine | -0.05026 | -0.1502 |
| nicotinamide | -0.10933 | 0.022752 |
| theobromine | 0.023504 | -0.06561 |
| 2,4-dihydroxypyrimidine-5-carboxylic acid | -0.11022 | 0.017263 |
| 5'-deoxyadenosine | -0.10864 | -0.01778 |
| D-pantothenic acid | 0.018393 | 0.048147 |
| purine | -0.10408 | -0.03557 |
| 2,4-dihydroxypteridine | -0.10175 | 0.070722 |
| uridine | -0.10558 | 0.014298 |
| urocanate | 0.030085 | -0.06916 |
| D--glucosamine | -0.008 | 0.013135 |
| adenosine | -0.10888 | 0.013041 |
| creatinine | -0.00604 | 0.018707 |
| 3',5'-cyclic AMP | 0.076786 | -0.03254 |
| D-glucono-1,5-lactone | -0.04115 | 0.072708 |
| 3-Ureidopropionate | -0.1043 | 0.016169 |
| adenosine 3',5'-cyclic monophosphate | 0.10362 | -0.0209 |
| tryptophan | -0.02784 | -0.16611 |
| L-kynurenine | 0.008284 | 0.16975 |
| phenylalanine | -0.09847 | -0.07594 |
| D-tryptophan | -0.02551 | -0.16835 |
| isoleucine | -0.00065 | -0.1732 |
| leucine | -0.01905 | -0.17611 |
| norleucine | -0.00334 | -0.17336 |
| 6'-hydroxynicotinate | -0.10862 | 0.020985 |
| N-acetyl-DL-gluatmic acid | -0.09686 | 0.028692 |
| 5-oxo-D-proline | -0.10427 | 0.03737 |
| acetylcholine | -0.10524 | 0.053916 |
| N-acetyl-L-cysteine | 0.069187 | -0.01911 |
| cytidine | -0.10875 | -0.00974 |
| cytidine 2'3'-cyclicmonophosphate | -0.0123 | 0.062747 |
| methionine | -0.10526 | 0.053171 |
| N-acetyl-D-glucosamine | 0.047606 | 0.15025 |
| 3-hydroxykynurenine | 0.024596 | -0.10337 |
| N-acetyl-D-galactosamine | -0.07254 | 0.038874 |
| 5-methylcytosine hydrochloride | -0.07615 | -0.08113 |
| uridine-5-monophosphate | 0.0521 | 0.10259 |
| cytosine | -0.10833 | -0.01368 |
| trigonelline | -0.04275 | 0.033348 |
| tyrosine | -0.11031 | -0.00143 |
| citramalate | -0.00848 | -0.15793 |
| betaine | -0.1079 | 0.00298 |
| proline | -0.11002 | 0.019804 |
| taurine | -0.00861 | -0.16129 |
| valine | -0.10572 | -0.04089 |
| pipecolate | -0.04711 | -0.12948 |
| D-aspartate | 0.070527 | 0.086588 |
| maleamate | -0.08158 | -0.10214 |
| 5,6-dihydrouracil | 0.088766 | -0.0202 |
| hypotaurine | -0.08136 | -0.0814 |
| cysteine | -0.04067 | 0.04276 |
| S-carboxymethyl-L-cysteine | -0.08365 | 0.010815 |
| 2R,3R---2,3-butanediol | -0.02397 | -0.14978 |
| glycolate positive mode | 0.059562 | -1.67E-05 |
| trans-4-hydroxyproline | 0.004849 | -0.17042 |
| cis-4-hydroxy-D-proline | 0.007273 | -0.1721 |
| glycine | 0.060851 | 0.015695 |
| mesoxalate | 0.080922 | 0.11976 |
| 2-amino-2-methyl propanoate | -0.10853 | 0.025973 |
| 3-aminoisobutanoate | 0.10298 | 0.016412 |
| alanine | 0.074059 | 0.099408 |
| diethanolamine | 0.1012 | 0.069036 |
| L-homocysteine thiolactone | 0.005487 | 0.097836 |
| L-norvaline | 0.007226 | 0.1624 |
| N-acetylglycine | 0.07412 | 0.11245 |
| N-amidino-L-aspartate | 0.073419 | 0.1194 |
| ethyl-3-ureidopropionate | 0.059776 | -0.11158 |
| N-acetyl-DL-serine | -0.0026 | -0.16696 |
| 3-sulfino-L-alanine | 0.036403 | -0.15444 |
| glyceraldehyde | 0.10093 | 0.047451 |
| maleimide | 0.10288 | -4.46E-05 |
| 1-methylnicotinamide | -0.007 | 0.096824 |
| 4-guanidinobutanoate | -0.09393 | 0.07356 |
| epinephrine | 0.053417 | 0.11214 |
| noradrenaline | 0.10095 | 0.025793 |
| octopamine | 0.093357 | 0.049665 |
| choline | -0.02829 | 0.022354 |
| N-acetylputrescine | 0.074107 | 0.0368 |
| N-formylglycine | -0.10936 | 0.01984 |
| 4-aminobutanoate | -0.10581 | 0.042447 |
| dopamine | 0.10093 | 0.047451 |
| D-alanine | -0.10171 | 0.054682 |
| ethanolamine | 0.077327 | 0.038585 |
| N-alpha-acetyl-L-lysine | -0.10928 | 0.028104 |
| creatine | 0.069442 | -0.09589 |
| 5-aminopentanoate | -0.1064 | -0.03621 |
| homoserine | 0.089976 | 0.094863 |
| L-carnitine | -0.08193 | 0.09837 |
| threonine | -0.10494 | 0.045486 |
| beta-alanine | 0.094435 | 0.079541 |
| L-allothreonine | -0.10562 | 0.050279 |
| SN-glycero-3-phosphocholine | 0.020026 | 0.1083 |
| 4-imidazoleacetic acid | -0.02167 | 0.10962 |
| 5aminoimidazole4carboxamide1betaDribofurano | -0.10608 | 0.028382 |
| deoxycarnitine | -0.09773 | 0.061332 |
| hydroxypyruvate | -0.08095 | -0.11946 |
| inosine 5'-monophosphate | -0.10497 | 0.05615 |
| 2-hydroxybutyric acid | -0.08055 | -0.1202 |
| coenzyme A | -0.11 | -0.0038 |
| glutamine | -0.10529 | 0.055555 |
| inosine 5'-triphosphate | -0.03598 | -0.16688 |
| serine | 0.034945 | 0.1181 |
| asparagine | -0.10943 | 0.020672 |
| D-glucosamine 6-sulfate | -0.09348 | 0.037887 |
| 2-acetamido-2-deoxy-beta-D-glucosylamine | 0.091763 | 0.058448 |
| pyridoxamine | -0.10773 | 0.030331 |
| glutamic acid | -0.10981 | 0.021569 |
| NAD | -0.1091 | 0.027973 |
| nepsilon,nepsilon,nepsilon,-trimethyllysine | -0.10937 | 0.020076 |
| carnosine | -0.03158 | -0.0657 |
| histidine | -0.11043 | 0.007303 |
| homocysteine | -0.01944 | -0.05438 |
| o-phospho-DL-serine | -0.05766 | -0.09623 |
| arginine | -0.11054 | 0.00703 |
| lysine | -0.11026 | 0.015277 |
| D-orinthine | -0.10967 | 0.014915 |
| L-orinthine | -0.10978 | 0.015515 |
| cadaverine | 0.082108 | -0.08687 |
| beta-nicotinamideadeninedinucleotidephosphate | -0.10974 | 0.020967 |
| guanosine 5'-diphosphoglucose | -0.00721 | 0.14466 |
| phosphocholine | -0.10937 | 0.016128 |
| methyl beta-D-galactoside | 0.075141 | -0.01008 |
| uracil | -0.09928 | 0.04099 |
| succinate | -0.10971 | 0.020707 |
| hypoxyxanthine | -0.04128 | -0.06502 |
| adenine | -0.10453 | -0.01869 |
| orotate | -0.09924 | -0.01298 |
| S-lactate | 0.057063 | -0.12207 |
| adenosine 2',3'-cyclic monophosphate | -0.10998 | 0.006149 |
| xanthosine | -0.10856 | 6.91E-05 |
| S-dihydroorotate | -0.07185 | -0.1262 |
| ascorbate | -0.10905 | 0.0144 |
| guanosine 3',5'-cyclic monophosphate | -0.04648 | -0.14155 |
| 3'-CMP | -0.10876 | 0.028134 |
| CMP | -0.1087 | 0.033573 |
| L-cystathionine | -0.10986 | 0.022059 |
| guanosine 5'-diphosphate | -0.09905 | 0.072111 |
| guanosine 5'-triphosphate | -0.06647 | 0.13801 |

Table S13. The loading factors for each individual metabolite from Figure S2-C.

| **Metabolite** | **LOADING 1** | **LOADING 2** |
| --- | --- | --- |
| uridine_5'-diphosphate | 0.022777 | -0.050236 |
| deoxyribose | 0.064908 | 0.10933 |
| alpha-hydroxyisobutyric_acid | 0.08093 | 0.044899 |
| homocystine | 0.10363 | 0.011167 |
| inosine | 0.091483 | -0.0095006 |
| isopentyl_pyrophosphate | 0.088529 | 0.094192 |
| D-glucose-6-phosphate | -0.068609 | -0.034743 |
| shikimate | 0.061907 | 0.099576 |
| tetrahydrofolate | 0.1038 | -0.027884 |
| D-glyceric_acid | 0.079555 | 0.074053 |
| inosine_5'-diphosphate | 0.10047 | -0.06183 |
| mevalolactone | 0.05072 | 0.099398 |
| D-lactose | 0.097082 | -0.054121 |
| paraxanthine | 0.039141 | 0.14532 |
| theophylline | 0.066306 | 0.090576 |
| 5'-methylthioadenosine | -0.09711 | 0.040061 |
| thymine | -0.0773 | 0.048688 |
| nicotinamide | 0.075826 | 0.081288 |
| theobromine | 0.068692 | 0.093548 |
| 2,4-dihydroxypyrimidine-5-carboxylic_acid | 0.086182 | 0.056008 |
| 5'-deoxyadenosine | -0.10602 | 0.014075 |
| D-pantothenic_acid | 0.10136 | 0.044906 |
| purine | -0.10318 | 0.021624 |
| 2,4-dihydroxypteridine | 0.057436 | 0.11523 |
| uridine | -0.10607 | -0.0004936 |
| urocanate | -0.047326 | 0.025474 |
| D--glucosamine | 0.095491 | 0.060219 |
| adenosine | -0.10488 | 0.012114 |
| creatinine | 0.053206 | 0.13903 |
| 3',5'-cyclic_AMP | -0.068518 | 0.1014 |
| D-glucono-1,5-lactone | -0.040179 | -0.025429 |
| 3-Ureidopropionate | -0.062719 | -0.054132 |
| adenosine_3',5'-cyclic_monophosphate | 0.0047388 | -0.01124 |
| tryptophan | -0.090009 | -0.050446 |
| L-kynurenine | 0.0095587 | -0.014527 |
| phenylalanine | -0.10084 | -0.038302 |
| D-tryptophan | -0.087829 | -0.051576 |
| isoleucine | -0.090787 | -0.083461 |
| leucine | -0.089867 | -0.068997 |
| norleucine | -0.091982 | -0.07415 |
| 6'-hydroxynicotinate | -0.10615 | 0.0091044 |
| N-acetyl-DL-gluatmic_acid | 0.029455 | 0.11381 |
| 5-oxo-D-proline | -0.096029 | 0.0055274 |
| acetylcholine | -0.081608 | 0.058779 |
| N-acetyl-L-cysteine | 0.078901 | -0.05435 |
| cytidine | -0.10546 | 0.012968 |
| cytidine_2'3'-cyclicmonophosphate | -0.0071141 | -0.053139 |
| methionine | -0.061557 | 0.14299 |
| N-acetyl-D-glucosamine | 0.095586 | 0.073112 |
| 3-hydroxykynurenine | 0.036583 | 0.0049802 |
| N-acetyl-D-galactosamine | -0.08505 | -0.056725 |
| 5-methylcytosine_hydrochloride | -0.10007 | 0.010603 |
| uridine-5-monophosphate | -0.092193 | -0.10272 |
| cytosine | -0.10534 | 0.013262 |
| trigonelline | 0.070851 | 0.082811 |
| tyrosine | -0.095756 | 0.082809 |
| citramalate | -0.051103 | -0.046734 |
| betaine | -0.030681 | 0.18588 |
| proline | 0.078833 | 0.077683 |
| taurine | -0.090052 | 0.07608 |
| valine | 0.057273 | -0.085473 |
| pipecolate | -0.10558 | -0.00027299 |
| D-aspartate | -0.063411 | -0.087155 |
| maleamate | -0.092691 | 0.020658 |
| 5,6-dihydrouracil | -0.088323 | 0.076637 |
| hypotaurine | -0.017499 | 0.056082 |
| cysteine | -0.064849 | -0.049809 |
| S-carboxymethyl-L-cysteine | 0.097364 | 0.040431 |
| 2R,3R---2,3-butanediol | -0.062667 | 0.1182 |
| glycolate_positive_mode | 0.091184 | -0.0078779 |
| trans-4-hydroxyproline | -0.0906 | -0.079683 |
| cis-4-hydroxy-D-proline | -0.090971 | -0.071094 |
| glycine | 0.075313 | -0.0025834 |
| mesoxalate | -0.087556 | 0.07565 |
| 2-amino-2-methyl_propanoate | -0.099195 | 0.066335 |
| 3-aminoisobutanoate | -0.098047 | 0.083999 |
| alanine | 0.072636 | 0.1484 |
| diethanolamine | 0.054567 | -0.14934 |
| L-homocysteine_thiolactone | -0.10538 | 0.023548 |
| L-norvaline | -0.10047 | 0.033846 |
| N-acetylglycine | -0.098927 | -0.005544 |
| N-amidino-L-aspartate | -0.043027 | 0.14318 |
| ethyl-3-ureidopropionate | -0.064998 | 0.010412 |
| N-acetyl-DL-serine | 0.017163 | 0.0035001 |
| 3-sulfino-L-alanine | 0.095757 | -0.030033 |
| glyceraldehyde | 0.10194 | -0.032853 |
| maleimide | -0.0035391 | -0.016203 |
| 1-methylnicotinamide | 0.065675 | 0.06805 |
| 4-guanidinobutanoate | -0.074543 | 0.14147 |
| epinephrine | -0.10058 | 0.015647 |
| noradrenaline | 0.0095217 | 0.13193 |
| octopamine | 0.036576 | 0.079615 |
| choline | 0.02984 | 0.037229 |
| N-acetylputrescine | -0.037274 | 0.17825 |
| N-formylglycine | -0.10603 | 0.01196 |
| 4-aminobutanoate | -0.097615 | 0.019711 |
| dopamine | 0.10194 | -0.032853 |
| D-alanine | 0.067133 | 0.13143 |
| ethanolamine | 0.064743 | 0.1392 |
| N-alpha-acetyl-L-lysine | -0.10627 | 0.01874 |
| creatine | -0.073565 | -0.090603 |
| 5-aminopentanoate | -0.1007 | 0.017597 |
| homoserine | -0.045095 | 0.13988 |
| L-carnitine | -0.011929 | 0.19483 |
| threonine | -0.069021 | -0.095378 |
| beta-alanine | -0.060544 | -0.067221 |
| L-allothreonine | -0.064878 | -0.13176 |
| SN-glycero-3-phosphocholine | -0.050622 | -0.0035331 |
| 4-imidazoleacetic_acid | -0.047167 | -0.0049347 |
| 5aminoimidazole4carboxamide1betaDribofurano | -0.1029 | 0.01986 |
| S-5'-adenosyl-L-homocysteine | 0.087699 | 0.034317 |
| deoxycarnitine | -0.070018 | 0.14955 |
| hydroxypyruvate | -0.046015 | -0.028067 |
| inosine_5'-monophosphate | -0.056773 | 0.10922 |
| 2-hydroxybutyric_acid | -0.033556 | -0.03317 |
| coenzyme_A | 0.04862 | 0.17459 |
| glutamine | -0.1018 | -0.012315 |
| inosine_5'-triphosphate | 0.045007 | -0.18691 |
| serine | 0.096015 | 0.026754 |
| asparagine | -0.10523 | 0.022486 |
| D-glucosamine_6-sulfate | -0.052299 | -0.030979 |
| 2-acetamido-2-deoxy-beta-D-glucosylamine | -0.061413 | -0.024067 |
| pyridoxamine | -0.10484 | -0.017797 |
| glutamic_acid | -0.10452 | 0.039646 |
| NAD | -0.016404 | 0.19854 |
| nepsilon,nepsilon,nepsilon,-trimethyllysine | -0.099366 | 0.066742 |
| carnosine | -0.0035615 | 0.048544 |
| histidine | -0.10674 | 0.0013895 |
| homocysteine | -0.044263 | -0.16626 |
| o-phospho-DL-serine | 0.073283 | -0.12212 |
| arginine | -0.10401 | 0.010428 |
| lysine | -0.10129 | 0.052667 |
| D-orinthine | -0.10222 | 0.041566 |
| L-orinthine | -0.10263 | 0.045951 |
| cadaverine | -0.098135 | 0.064243 |
| beta-nicotinamideadeninedinucleotidephosphate | -0.083066 | 0.10181 |
| guanosine_5'-diphosphoglucose | -0.10284 | 0.050188 |
| phosphocholine | -0.10556 | 0.021526 |
| methyl_beta-D-galactoside | 0.07642 | 0.125 |
| uracil | 0.057789 | 0.12954 |
| succinate | -0.10062 | 0.067442 |
| hypoxyxanthine | -0.10222 | 0.034778 |
| adenine | -0.096738 | 0.032737 |
| orotate | -0.1057 | 0.030245 |
| S-lactate | -0.057715 | -0.05086 |
| adenosine_2',3'-cyclic_monophosphate | -0.10371 | 0.0030723 |
| xanthosine | -0.09364 | 0.043358 |
| S-dihydroorotate | -0.023338 | 0.015539 |
| ascorbate | 0.10643 | -0.024794 |
| guanosine_3',5'-cyclic_monophosphate | -0.08288 | -0.044793 |
| 3'-CMP | -0.082778 | 0.10251 |
| DAMP | 0.039452 | 0.1047 |
| CMP | -0.086516 | 0.081927 |
| L-cystathionine | -0.0728 | 0.14576 |
| guanosine_5'-diphosphate | 0.089185 | 0.10182 |
| guanosine_5'-triphosphate | 0.065848 | 0.022032 |

Table S14. The loading factors for each individual metabolite from Figure S2-D.

| **Metabolite** | **LOADING 1** | **LOADING 2** |
| --- | --- | --- |
| uridine_5'-diphosphate | -0.013539 | 0.16633 |
| deoxyribose | 0.092733 | 0.019286 |
| alpha-hydroxyisobutyric_acid | 0.099517 | 0.062382 |
| homocystine | 0.0041777 | 0.16929 |
| inosine | -0.0016649 | 0.16686 |
| isopentyl_pyrophosphate | 0.019653 | 0.16353 |
| D-glucose-6-phosphate | -0.067453 | 0.045622 |
| shikimate | 0.077603 | 0.066618 |
| tetrahydrofolate | 0.0029058 | 0.16847 |
| D-glyceric_acid | 0.071616 | 0.063016 |
| inosine_5'-diphosphate | 0.00072935 | 0.16392 |
| mevalolactone | -0.051875 | 0.14388 |
| D-lactose | -0.0014818 | 0.16845 |
| paraxanthine | -0.073545 | 0.10243 |
| theophylline | 0.075065 | 0.060963 |
| 5'-methylthioadenosine | -0.10579 | 0.058291 |
| thymine | -0.10772 | 0.016973 |
| nicotinamide | 0.08089 | 0.030327 |
| theobromine | 0.054339 | 0.081629 |
| 2,4-dihydroxypyrimidine-5-carboxylic_acid | -0.1144 | 0.0062152 |
| 5'-deoxyadenosine | -0.11417 | -0.0036263 |
| D-pantothenic_acid | -0.047976 | 0.013705 |
| purine | -0.095441 | -0.021521 |
| 2,4-dihydroxypteridine | 0.030718 | -0.012699 |
| uridine | -0.10756 | -0.015288 |
| urocanate | 0.064213 | 0.14055 |
| D--glucosamine | -0.024017 | 0.16258 |
| adenosine | -0.10554 | -0.031578 |
| creatinine | 0.010563 | -0.081357 |
| 3',5'-cyclic_AMP | 0.073044 | -0.016793 |
| D-glucono-1,5-lactone | -0.06329 | 0.10876 |
| 3-Ureidopropionate | -0.039541 | 0.097985 |
| adenosine_3',5'-cyclic_monophosphate | 0.093712 | -0.037653 |
| tryptophan | 0.0264 | -0.14139 |
| L-kynurenine | 0.028994 | -0.079348 |
| phenylalanine | -0.097477 | -0.079646 |
| D-tryptophan | 0.024636 | -0.14231 |
| isoleucine | -0.084974 | -0.1041 |
| leucine | -0.077074 | -0.11697 |
| norleucine | -0.081295 | -0.1143 |
| 6'-hydroxynicotinate | -0.094702 | 0.027492 |
| N-acetyl-DL-gluatmic_acid | -0.060208 | 0.14272 |
| 5-oxo-D-proline | 0.032665 | 0.13396 |
| acetylcholine | -0.104 | 0.005722 |
| N-acetyl-L-cysteine | 0.081705 | -0.01791 |
| cytidine | -0.10875 | -0.020662 |
| cytidine_2'3'-cyclicmonophosphate | 0.10855 | -0.042771 |
| methionine | -0.068599 | 0.022847 |
| N-acetyl-D-glucosamine | 0.04017 | 0.057195 |
| 3-hydroxykynurenine | -0.0052043 | 0.082045 |
| N-acetyl-D-galactosamine | -0.07431 | 0.088675 |
| 5-methylcytosine_hydrochloride | 0.052129 | 0.13104 |
| uridine-5-monophosphate | -0.0012472 | 0.073547 |
| cytosine | -0.10876 | -0.017224 |
| trigonelline | 0.10537 | 0.06817 |
| tyrosine | -0.11287 | 0.014456 |
| citramalate | -0.038348 | 0.089759 |
| betaine | -0.11197 | 0.034327 |
| proline | -0.11414 | 0.0089089 |
| taurine | 0.00054594 | 0.16492 |
| valine | -0.019383 | 0.15594 |
| pipecolate | 0.029834 | 0.073163 |
| D-aspartate | 0.11072 | -0.038341 |
| maleamate | -0.099656 | 0.017687 |
| 5,6-dihydrouracil | 0.0032549 | 0.12069 |
| hypotaurine | -0.01644 | 0.072133 |
| cysteine | -0.006639 | 0.15913 |
| S-carboxymethyl-L-cysteine | -0.1095 | 0.031356 |
| 2R,3R---2,3-butanediol | -0.079504 | -0.016211 |
| glycolate_positive_mode | 0.082838 | 0.11763 |
| trans-4-hydroxyproline | -0.089124 | -0.10138 |
| cis-4-hydroxy-D-proline | -0.079793 | -0.11298 |
| glycine | 0.056169 | 0.141 |
| mesoxalate | -0.11224 | 0.025153 |
| 2-amino-2-methyl_propanoate | -0.11448 | 0.014748 |
| 3-aminoisobutanoate | -0.11287 | 0.019771 |
| alanine | -0.11384 | -0.014433 |
| diethanolamine | 0.091052 | 0.085892 |
| L-homocysteine_thiolactone | -0.068506 | 0.012879 |
| L-norvaline | -0.10459 | 0.010181 |
| N-acetylglycine | 0.081853 | 0.063188 |
| N-amidino-L-aspartate | -0.010644 | -0.077007 |
| ethyl-3-ureidopropionate | -0.030816 | 0.052102 |
| N-acetyl-DL-serine | -0.062322 | -0.016293 |
| 3-sulfino-L-alanine | 0.10078 | 0.042702 |
| glyceraldehyde | 0.097145 | 0.071719 |
| maleimide | 0.046948 | 0.079376 |
| 1-methylnicotinamide | 0.09233 | -0.012822 |
| 4-guanidinobutanoate | -0.10159 | 0.073389 |
| epinephrine | 5.52E-06 | 0.048685 |
| noradrenaline | 0.049463 | -0.073591 |
| octopamine | 0.09122 | -0.035569 |
| choline | 0.051384 | 0.1092 |
| N-acetylputrescine | 0.074025 | -0.02084 |
| N-formylglycine | -0.11387 | 0.019959 |
| 4-aminobutanoate | -0.11385 | 0.020508 |
| dopamine | 0.097145 | 0.071719 |
| D-alanine | -0.11431 | -0.00041581 |
| ethanolamine | -0.027264 | 0.12308 |
| N-alpha-acetyl-L-lysine | -0.038093 | 0.11178 |
| creatine | -0.092605 | -0.070296 |
| 5-aminopentanoate | -0.11442 | -0.0097613 |
| homoserine | -0.11426 | -0.0037353 |
| L-carnitine | -0.087236 | 0.064149 |
| threonine | -0.11119 | 0.032006 |
| beta-alanine | -0.024739 | 0.073252 |
| L-allothreonine | -0.10961 | 0.035717 |
| SN-glycero-3-phosphocholine | 0.016975 | 0.033916 |
| 4-imidazoleacetic_acid | 0.055893 | 0.027064 |
| 5aminoimidazole4carboxamide1betaDribofurano | -0.09032 | -0.027065 |
| S-5'-adenosyl-L-homocysteine | 0.069458 | 0.0099361 |
| deoxycarnitine | -0.094856 | 0.069391 |
| hydroxypyruvate | -0.030733 | 0.077079 |
| inosine_5'-monophosphate | 0.095693 | 0.054049 |
| 2-hydroxybutyric_acid | -0.024791 | 0.076923 |
| coenzyme_A | -0.10217 | 0.055358 |
| glutamine | -0.10959 | 0.0098989 |
| inosine_5'-triphosphate | -0.0046657 | 0.017685 |
| serine | -0.070034 | 0.092923 |
| asparagine | 0.11363 | 0.0044299 |
| D-glucosamine_6-sulfate | -0.10194 | 0.064274 |
| 2-acetamido-2-deoxy-beta-D-glucosylamine | -0.024313 | 0.11887 |
| pyridoxamine | -0.11054 | 0.01863 |
| glutamic_acid | 0.015409 | 0.10539 |
| NAD | 0.05746 | 0.13402 |
| nepsilon,nepsilon,nepsilon,-trimethyllysine | 0.1088 | 0.039919 |
| carnosine | -0.083138 | 0.042872 |
| histidine | -0.081091 | 0.044599 |
| homocysteine | -0.085216 | 0.088685 |
| o-phospho-DL-serine | 0.039794 | 0.14745 |
| arginine | -0.10203 | 0.041661 |
| lysine | -0.092494 | 0.07479 |
| D-orinthine | -0.11087 | 0.023537 |
| L-orinthine | -0.11085 | 0.025583 |
| cadaverine | -0.094139 | -0.00042961 |
| beta-nicotinamideadeninedinucleotidephosphate | -0.10433 | 0.052832 |
| guanosine_5'-diphosphoglucose | 0.03348 | 0.12379 |
| phosphocholine | -0.11019 | 0.042751 |
| methyl_beta-D-galactoside | 0.066215 | -0.0028653 |
| uracil | 0.11199 | 0.030913 |
| succinate | -0.10756 | 0.038752 |
| hypoxyxanthine | 0.083845 | 0.022016 |
| adenine | -0.087196 | 0.034275 |
| orotate | -0.061507 | -0.024896 |
| S-lactate | 0.015756 | -0.099189 |
| adenosine_2',3'-cyclic_monophosphate | -0.11295 | -0.0042673 |
| xanthosine | -0.067501 | 0.0023531 |
| S-dihydroorotate | -0.043742 | 0.014241 |
| ascorbate | 0.10304 | 0.057087 |
| guanosine_3',5'-cyclic_monophosphate | 0.084104 | -0.090104 |
| 3'-CMP | 0.094526 | 0.056835 |
| DAMP | -0.091554 | 0.035181 |
| CMP | 0.097389 | 0.048618 |
| L-cystathionine | -0.11456 | 0.012889 |
| guanosine_5'-diphosphate | 0.033626 | 0.15272 |
| guanosine_5'-triphosphate | -0.012024 | -0.048711 |
